# Synthesis and Characterization of Tetraphenylethene AIEgen-Based Push–Pull Chromophores for Photothermal Applications: Could the Cycloaddition–Retroelectrocyclization Click Reaction Make Any Molecule Photothermally Active?

**DOI:** 10.3390/ijms24108715

**Published:** 2023-05-13

**Authors:** Maxime Roger, Yann Bretonnière, Yann Trolez, Antoine Vacher, Imane Arbouch, Jérôme Cornil, Gautier Félix, Julien De Winter, Sébastien Richeter, Sébastien Clément, Philippe Gerbier

**Affiliations:** 1 ICGM, CNRS UMR 5253, ENSCM, University of Montpellier, 34293 Montpellier, France; maxime.roger@univ-angers.fr (M.R.); gautier.felix@umontpellier.fr (G.F.); sebastien.richeter@umontpellier.fr (S.R.); 2 ENS de Lyon, CNRS UMR 5182, Laboratoire de Chimie, University of Lyon, 69364 Lyon, France; yann.bretonniere@ens-lyon.fr; 3 Ecole Nationale Supérieure de Chimie de Rennes, CNRS, ISCR—UMR 6226, University of Rennes, 35065 Rennes, France; yann.trolez@ensc-rennes.fr (Y.T.); antoine.vacher@univ-rennes1.fr (A.V.); 4 Laboratory for Chemistry of Novel Materials, University of Mons-UMONS, 7000 Mons, Belgium; imane.arbouch@umons.ac.be (I.A.); jerome.cornil@umons.ac.be (J.C.); 5 Organic Synthesis and Mass Spectrometry Laboratory (S2MOs), University of Mons-UMONS, 7000 Mons, Belgium; julien.dewinter@umons.ac.be

**Keywords:** TPE, AIEgen, cycloaddition-retroelectrocyclization, click chemistry, photothermal applications

## Abstract

Three new tetraphenylethene (TPE) push–pull chromophores exhibiting strong intramolecular charge transfer (ICT) are described. They were obtained via [2 + 2] cycloaddition–retroelectrocyclization (CA-RE) click reactions on an electron-rich alkyne-tetrafunctionalized TPE (TPE-alkyne) using both 1,1,2,2-tetracyanoethene (TCNE), 7,7,8,8-tetracyanoquinodimethane (TCNQ) and 2,3,5,6-tetrafluoro-7,7,8,8-tetracyanoquinodimethane (F_4_-TCNQ) as electron-deficient alkenes. Only the starting TPE-alkyne displayed significant AIE behavior, whereas for TPE-TCNE, a faint effect was observed, and for TPE-TCNQ and TPE-F_4_-TCNQ, no fluorescence was observed in any conditions. The main ICT bands that dominate the UV–Visible absorption spectra underwent a pronounced red-shift beyond the near-infrared (NIR) region for TPE-F_4_-TCNQ. Based on TD-DFT calculations, it was shown that the ICT character shown by the compounds exclusively originated from the clicked moieties independently of the nature of the central molecular platform. Photothermal (PT) studies conducted on both TPE-TCNQ and TPE-F4-TCNQ in the solid state revealed excellent properties, especially for TPE-F4-TCNQ. These results indicated that CA-RE reaction of TCNQ or F_4_-TCNQ with donor-substituted are promising candidates for PT applications.

## 1. Introduction

**Scheme 1:**
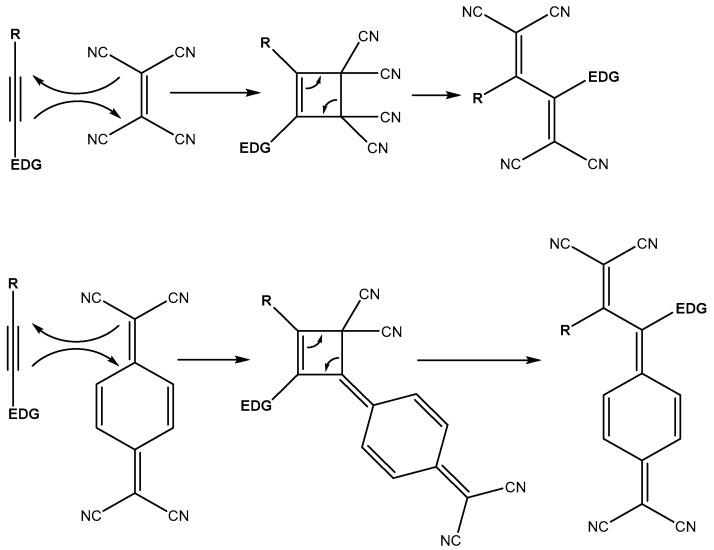
Mechanism of the CA-RE click reaction between TCNE (**top**) or TCNQ (**bottom**) and alkynes activated by an electron-donor group (EDG). When TCNQ is used, the quinoidal moiety is located next to the EDG group [54,82].

Although the phenomenon has been known and briefly described before [1], it was Ben Zhong Tang who first described and tried to explain the unusual behavior of 1-methyl-1,2,3,4,5-pentaphenylsilole, which was not luminescent in solution in a good solvent but became highly emissive in the solid state [2]. This very rare behavior, called Aggregation-Induced Emission (AIE), is opposed to the behavior classically encountered in fluorophores with a planar and rigid structure, which are generally fluorescent in solution and non-emissive in the solid state (Aggregation-Caused Quenching—ACQ). Following this work, in just over 20 years, the number of publications concerning the description of new AIE luminogens (AIEgens) has increased exponentially [3], and the the mechanisms leading to the observation of this phenomenon are now well known [4,5,6]. The considerable success of the AIE phenomenon lies in the fact that it makes possible it to effectively address a major issue, which is the emission in the solid or aggregated state of molecules, a problem of the utmost importance for many scientific fields or technological applications [4,7,8]. Among the many areas taking advantage of AIEgens, we can mention optoelectronics with OLEDs [9,10], stimuli-responsive materials [11,12,13], anticounterfeiting or encryption [12,14], sensing [15,16,17] and biology or medicine, fields in which the use of AIEgens has enabled spectacular advances [18,19,20,21,22,23]. Indeed, AIEs are used in the fields of imaging [24,25], elucidation of biological processes [26,27,28], innovative therapies such as photodynamic therapy (PDT) [29,30,31], photothermal therapy (PTT) [32,33], sonodynamic therapy enhancement [34], cocktail therapy [35] and theranostics [36,37]. Due to the specificity of biological applications, it is necessary to trigger the AIEgens with light in the near-infrared (NIR) domain, which has the advantage on the one hand of not being harmful to the cells, and on the other hand of having a good depth of penetration into the living tissues [38,39]. As a result, it was necessary to substantially reduce the HOMO–LUMO energy gap (E_g_) of conventional AIEgens whose absorption bands are located in the UV range. Different strategies can be considered to increase the excitation and emission wavelengths of the fluorophores. The first one consists of increasing the conjugation length of the π-system. However, this strategy is rapidly discarded due to solubility issues, which require “decorating” the molecules with flexible chains. The second one consists of introducing electron-attracting (A) and/or donating (D) groups into the π-conjugated system [40,41,42]. Such groups induce changes in orbital energy levels, either an increase in HOMO energy level (influence of donor group), or a decrease in LUMO energy level (influence of attracting group) of the molecule leading to a narrowing of the E_g_ [43]. Among the attractor groups yielding the best results are the dicyanovinyl groups. For instance, the latter can be introduced via a Knoevenagel condensation reaction of malonitrile on an AIEgen functionalized with a carbonyl [39,44]. Another approach consists of taking advantage of the “click-chemistry” introduced by K. B. Sharpless, which is effective in introducing various groups under mild, catalyst-free and atom-saving conditions [45]. More especially, the [2 + 2] cycloaddition–retroelectrocyclizations (CA-RE) that involve the reaction between an electron-rich alkyne and an electron-deficient alkene (Scheme 1) have recently attracted significant attention [46,47]. The CA-RE reaction was first reported by M. I. Bruce in 1981 in the field of organometallic chemistry by reacting 1,1,2,2-tetracyanoethene (TCNE) with metallocene-substituted acetylides [48]. It was 18 years later that it was used for the synthesis of DA-type thiophenevinylidene derivatives for nonlinear optical (NLO) applications in the groups of A. K.-Y. Jen and U. W. Suter [49,50]. This reaction has been widely exploited and studied by the group of F. Diederich to develop a new platform of simple molecules with nonlinear optical (NLO) properties [51,52]. In the continuation of these works, he showed that it was possible to modulate the optical and electrochemical properties of the clicked compounds by extending the CA-RE reaction to other electron-deficient alkenes which have a similar reactivity, such as 7,7,8,8-tetracyanoquinodimethane (TCNQ) and 2,3,5,6-tetrafluoro-7,7,8,8-tetracyanoquinodimethane (F_4_-TCNQ) [53,54,55]. Since then, researchers have used CA-RE to synthesize D-π-A push–pull systems to be used in various applications such as optoelectronics [56,57,58,59,60,61,62,63,64,65,66,67,68], NLO [69,70,71], photoacoustic (PA) imaging [72,73,74,75,76] and theranostics [77]. Surprisingly, although the photoacoustic and photothermal (PT) phenomena are related, very few papers describe the use of CA-RE clicked products for PT applications [72,78].

In this work, we used the [2 + 2] CA-RE reaction with the aim of obtaining new AIE-based PT materials displaying a strong charge transfer character. We chose the AIEgen archetypal tetraphenylethene (TPE) as the central core due to its very good ability to retain its AIE properties, although it is incorporated in more complicated molecular architectures [79,80]. Moreover, when linked to naphthalene diimide-fused 2-(1,3-dithiol-2-ylidene)acetonitrile derivatives, it has already shown its ability to lead to AIE-based PT materials [81]. In order to achieve the CA-RE reactions, we functionalized the four arms of the TPE with C≡C triple bonds capped by dialkylanilino moieties as electron-donor groups. The resulting clicked products with TCNE, TCNQ and F_4_-TCNQ showed a pronounced CT characterized by an NIR strong absorption, in particular, for TPE-TCNQ and TPE-F_4_-TCNQ, in the biological transparency window (NIR-I, 750–1000 nm). These two latter products were found to possess good thermal properties, allowing them to display rather good photothermal performances.

## 2. Results and Discussion

### 2.1. Synthesis

**Scheme 2:**
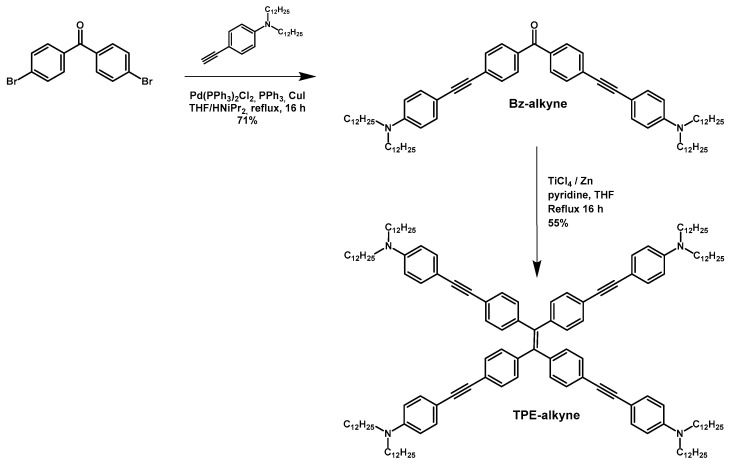
Reaction pathway towards TPE-alkyne.

The preparation of compounds TPE-TCNE, TPE-TCNQ and TPE-F_4_-TCNQ started with TPE-alkyne, whose synthesis involved a McMurry homocoupling reaction of the benzophenone derivative Bz-alkyne with a yield of 55% (Scheme 2). Bz-alkyne was synthesized in 71% yield by using a Sonogashira cross-coupling reaction between 4,4′-dibromobenzophenone and *N*,*N*’-didodecyl-4-ethynylaniline. All attempts to obtain TPE-alkyne through a Sonogashira cross-coupling reaction between 1,1,2,2-tetrakis(4-bromophenyl)ethene and *N*,*N*’-didodecyl-4-ethynylaniline invariably conducted to inseparable mixtures of diversely substituted compounds.

**Scheme 3:**
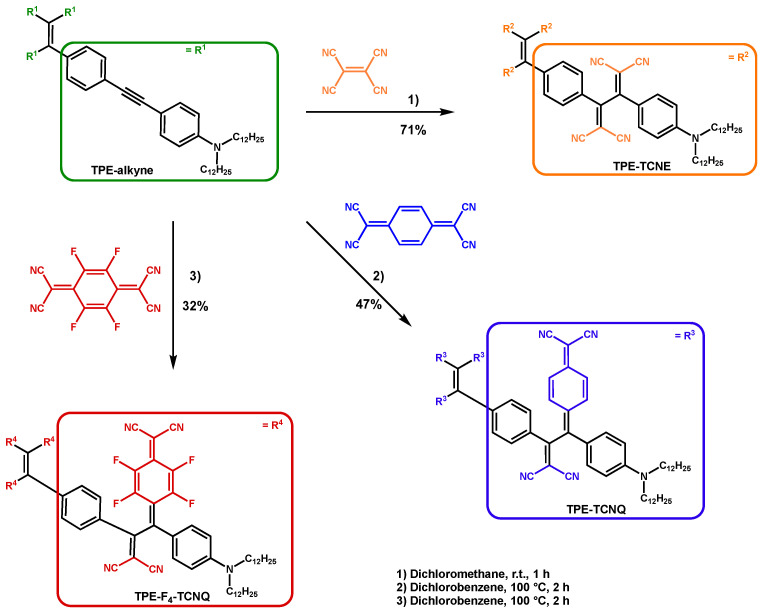
Synthetic scheme of compounds TPE-alkyne, TPE-TCNQ and TPE-F_4_-TCNQ.

Finally, [2 + 2] CA-RE reactions were conducted by using a 50% excess of tetracyano derivatives (TCNE, TCNQ and F4-TCNQ) in various solvents at room temperature to form TPE-TCNE, TPE-TCNQ and TPE-F_4_-TCNQ as dark film-forming solids after purification with column chromatography on silica gel (Scheme 3).

**Figure 1:**
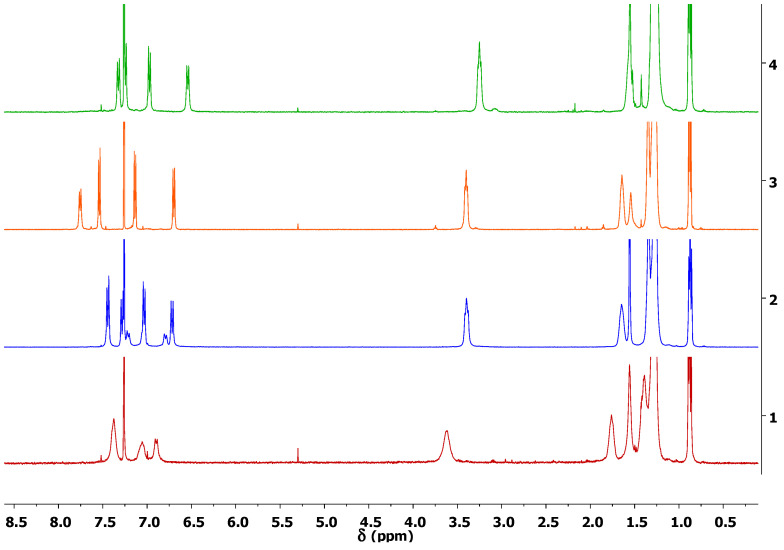
^1^H NMR spectra of TPE-alkyne (green), TPE-TCNE (orange), TPE-TCNQ (blue) and TPE-F_4_-TCNQ (red) recorded in CDCl_3_.

**Figure 2:**
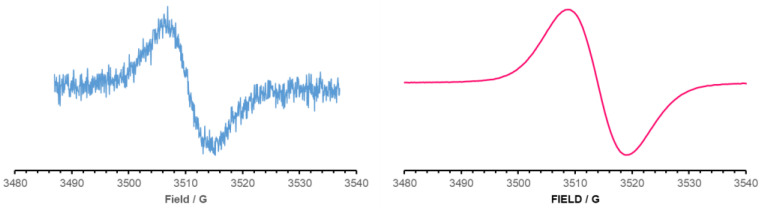
ESR spectra of TPE-F_4_-TCNQ in THF solution (**right**) and in the solid state (**left**), recorded at room temperature.

All the compounds were fully characterized by ^1^H, ^13^C{^1^H}, ^19^F{^1^H} NMR spectroscopy and high-resolution mass spectrometry (HR-MS) (see ESI for spectral details). Examining their ^1^H NMR spectra, some interesting points can be noted (Figure 1): (i) as expected, the grafting of electron-withdrawing groups on the triple bond led to a deshielding of the protons located in their vicinity (doublets in the aromatic region), as well as those of the methylene groups bonded to the nitrogen atoms of the aniline moieties (δ = 3.25 − 3.55 ppm); (ii) the comparison of the chemical shifts of the aromatic protons between TPE-alkyne, TPE-TCNE and TPE-TCNQ highlighted the localization of the dicyanoquinomethanyl moiety on the aniline side in agreement with previous findings [54]; (iii) due to the highly twisted conformation of the butadiene in TPE-TCNQ (see ESI), all the protons present in the quinoid ring are not equivalents, giving rise to four doublets among which two are clearly seen in Figure 1 (see ESI for more details) and *iv*) the spectrum obtained for TPE-F_4_-TCNQ is poorly resolved, implying the presence of paramagnetic species as observed elsewhere [46,53]. Indeed, the ESR spectrum of a THF solution of TPE-F_4_-TCNQ showed an unresolved weak signal with a *g* factor of 2.0027 and a band width of 7.50 G, whereas, in the solid state, a much more intense signal with a *g* factor of 2.0021 and a band width of 10.25 G was observed (Figure 2).

### 2.2. Photophysical Properties

**Figure 3:**
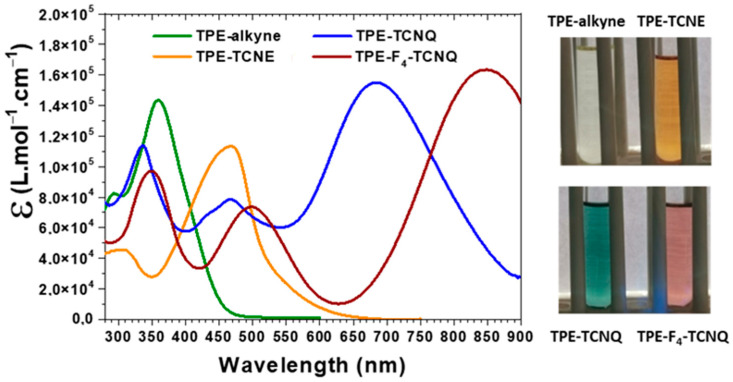
UV–Visible absorption spectra of investigated compounds in THF solutions (C = 6 × 10^−6^ M) (**left**) and images of their corresponding solutions (**right**).

**Figure 4:**
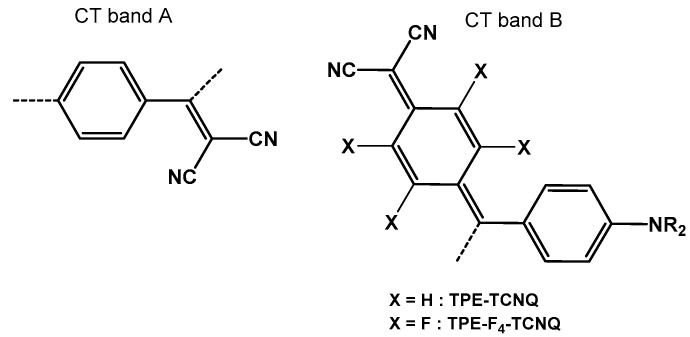
Chromophores likely involved in the CT bands A and B.

The UV–Visible absorption spectra of the investigated compounds are shown in Figure 3. The starting compound TPE-alkyne only showed a major absorption peak at 359 nm corresponding to a π-π* transition (vide supra). Contrastingly, the spectra of TPE-TCNE, TPE-TCNQ and TPE-F_4_-TCNQ showed additional intense absorption peaks in the visible–NIR region (see Table 1 for data). These peaks possessed strong molar extinction coefficients, likely corresponding to intramolecular charge transfer (ICT) absorptions involving the terminal amino groups and dicyanovinylic moieties [76]. Moreover, as observed previously, the effect of the increasing electron-withdrawing strength of the adduct that is F_4_-TCNQ > TCNQ > TCNE is witnessed by a marked bathochromic shift in the CT absorption [70,71,72,83,84,85,86]. Besides the intense charge transfer band involving the dicyanoquinoïd moieties (CT band B) observed above 600 nm for TPE-TCNQ and TPE-F_4_-TCNQ, the less intense CT band (CT band A) that was observed for all the clicked compounds in the 450–550 nm region involved the dicyanovinylic moieties (see Figure 4) [76]. Finally, although this is commonly observed for compounds exhibiting a charge transfer phenomenon, we have not demonstrated solvatochromism for these TPE adducts [66]. This may be related to the symmetry of these molecules, which implies that they have a weak total dipolar moment in the excited state. The absorbance spectra of all adducts deposited as thin films on quartz slides were like those recorded in THF solutions (Figure S23). As seen in Table 1, the longer wavelength absorptions experienced moderate (TPE-TCNE: 7 nm and TPE-4F-TCNQ: 15 nm) to appreciable (TPE-TCNQ: 50 nm) red shifts in the solid state.

**Figure 5:**
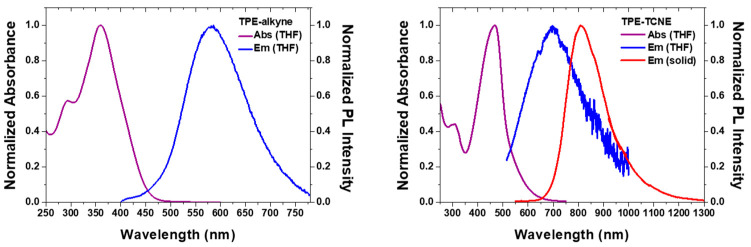
UV–Visible absorption and fluorescence spectra of TPE-alkyne (**left**) and TPE-TCNE (**right**) recorded in solution and in the solid state.

**Figure 6:**
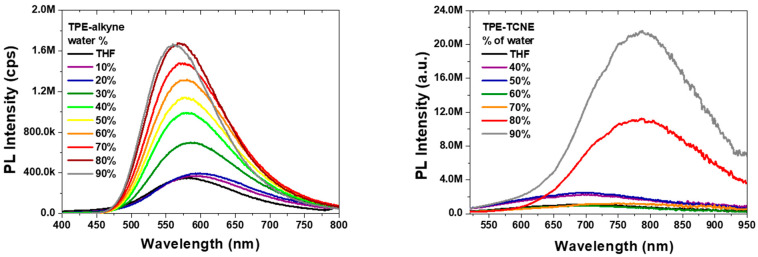
Evolution of the emission spectra as a function of water content in THF solutions for TPE-alkyne (**left**) and TPE-TCNE (**right**, the emission spectra recorded with 20 and 30% of water are strictly the same as those recorded at 0 and 40%).

The fluorescence spectra were also recorded both in solution (THF) and in the aggregated state for all compounds. As observed in most cases with CA-RE products [87,88,89], only the TPE-alkyne precursor exhibited appreciable fluorescence with an emission maximum at 582 nm and a quantum yield of 13%. A faint fluorescence was detected for TPE-TCNE with an emission maximum at ca. 700 nm and a quantum yield lower than 1%. The corresponding spectra are shown in Figure 5. Interestingly, the emission maximum showed a dependence on the pH of the solution with a red-shift of 44 nm in acidic medium (see Figure S26). As observed with most TPE derivatives, the emission intensity increased markedly when going from the solution to the aggregated state, indicating an AIE behavior [79]. Consequently, we decided to follow the evolution of the emission spectra of both TPE-alkyne and TPE-TCNE in various mixtures of THF/water, water being a bad solvent for these compounds that should induce an aggregation phenomenon at a certain percentage. As shown in Figure 6, the emission intensity gradually increased as the water percentage exceeded 20% for TPE-alkyne, and more abruptly above 70% for TPE-TCNE, indicating that the presence of the dicyanovinylic moieties induced an increase in water solubility. Interestingly, both compounds exhibited an opposite shift when passing from the solvated to the aggregated state, with TPE-alkyne experiencing a slight blue-shift, whereas for TPE-TCNE, a more pronounced red-shift was noticed.

### 2.3. Electrochemistry

**Figure 7:**
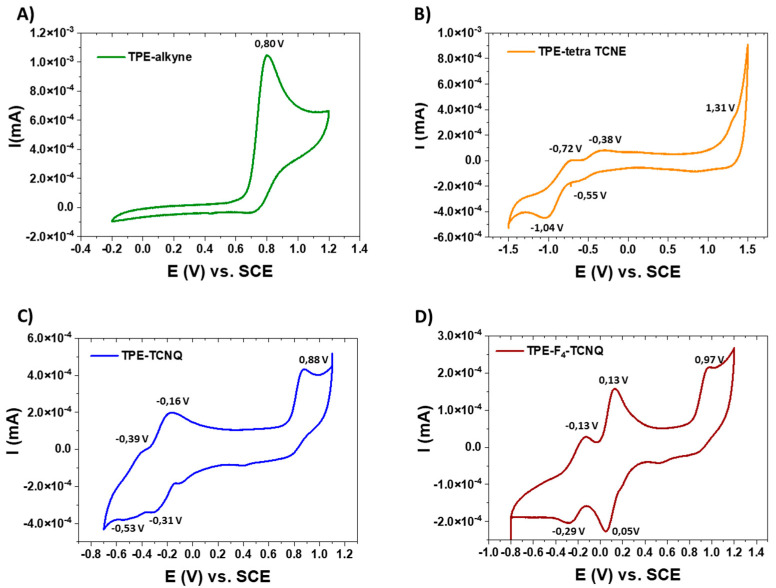
Cyclic voltammograms of TPE-alkyne (**A**), TPE-TCNE (**B**), TPE-TCNQ (**C**) and TPE-F_4_-TCNQ (**D**) in CH_2_Cl_2_ + Bu_4_NPF_6_ solutions. Scan rate: 100 mV.s^−1^, Pt disk working electrode. Potentials are given vs. SCE.

To evaluate the electronic properties of the different cycloadducts, their cyclic voltammograms (CVs) were recorded as along with the CV of the TPE-alkyne precursor for comparison (see Figure 7). This latter only showed one irreversible oxidation wave at around 0.80 V vs. SCE (peak potential), which could be attributed to the oxidation of the four anilines. One can notice that the four oxidations occurred at the same potential, probably because of the low conjugation between anilines due to the twisted geometry around the central TPE core. This irreversible oxidation was also observed in the case of cycloadducts, but it was shifted to higher potentials (peak potentials at 1.31 V vs. SCE for TPE-TCNE, 0.88 V vs. SCE for TPE-TCNQ and 0.97 V vs. SCE for TPE-F_4_-TCNQ). This may be attributed to the presence of the strong electron acceptors conjugated to the anilines, rendering their oxidations more difficult. By contrast with the TPE-alkyne precursor, two reversible reduction waves were also observed. The reduction potentials strongly depend on the nature of the electron-accepting groups. In the case of TPE-TCNE, the two reductions occurred at −0.45 and −0.88 V vs. SCE (these potentials were calculated as the half-sum of the anodic and cathodic potential peaks, see the note in Table 2), which are in line with previously reported values for this kind of tetracyanobutadiene (TCBD) [52]. TPE-TCNQ was easier to reduce with reductions at −0.23 and −0.46 V vs. SCE due to a higher delocalization of the newly formed negative charges thanks to the dicyanoquinodimethane moiety. The reduction potentials were even higher in the case of TPE-F_4_-TCNQ: +0.09 and −0.21 V vs. SCE due to the significant strengthening of the electron-accepting ability of the dicyanoquinodimethane moiety due to the presence of the fluorine atoms [54]. This particularly high first reduction potential may explain the “spontaneous” reduction of the system that generates the above-mentioned ESR signal in solution and in the solid state.

### 2.4. Electronic Structure Calculations

To gain more insights into the photophysical and electrochemical measurements on the four TPE derivatives, theoretical calculations based on (Time-Dependent) Density Functional Theory (TD)-DFT were performed [90,91,92,93,94]. All calculations were carried out using the long-range hybrid CAM-B3LYP functional [95] and a 6-311G (d,p) basis set, as motivated by the fact that the DFT functionals with long-range corrections better describe excited states with a pronounced charge transfer character [96].

**Figure 8:**
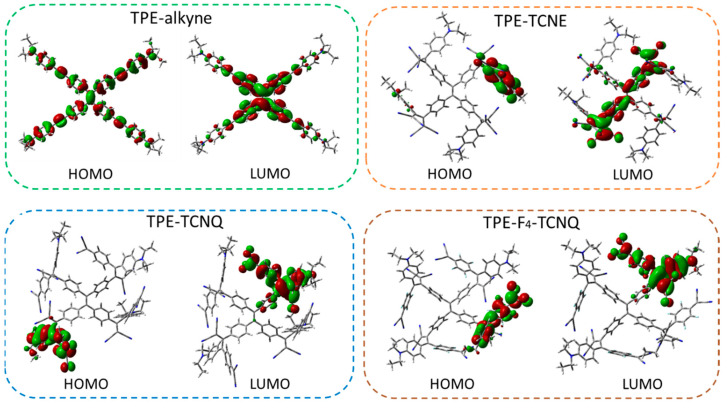
Frontier orbitals of the investigated compounds from the CAM-B3LYP/6.311g(d,p)-calculated optimized structures. Isovalues are set to 0.02 e/au^3^.

The shape of the frontier orbitals in the optimized structures obtained from the DFT calculations are displayed in Figure 8 (see also Figure S44). For TPE-alkyne, the HOMO is delocalized evenly on the entire molecule while the LUMO is denser on the TPE core. In TPE-TCNE, the HOMO is localized on the extremity of one TCBD arm following the introduction of an electron-accepting group in its center, thus breaking apart the large delocalization observed for the pristine compound. Similarly, the HOMO-1 to HOMO-3 levels get localized over the other branches (Figure S46). The LUMO spreads along a diagonal involving both the TPE core and the inner part of two TCBD arms. A similar pattern prevails for the LUMO + 1, while the LUMO + 2 and LUMO + 3 are delocalized over the other diagonal (Figure S46). In the case of TPE-TCNQ and TPE-F_4_-TCNQ, the frontier orbital spatial distribution is quite similar, with the HOMO and LUMO levels localized over the terminal donor part and inner acceptor part, respectively, of a single arm. The deeper occupied and higher unoccupied levels are localized over the other branches. Here, the TPE core is thus weakly involved in the frontier orbital distribution. Excited states with a pronounced intra- and/or interbranch character are thus expected at low energy. Since the molecular orbitals are not uniformly distributed over all branches, these frontier molecular orbitals are quasi-degenerate, particularly the HOMOs (see Figures S45 and S46).

**Figure 9:**
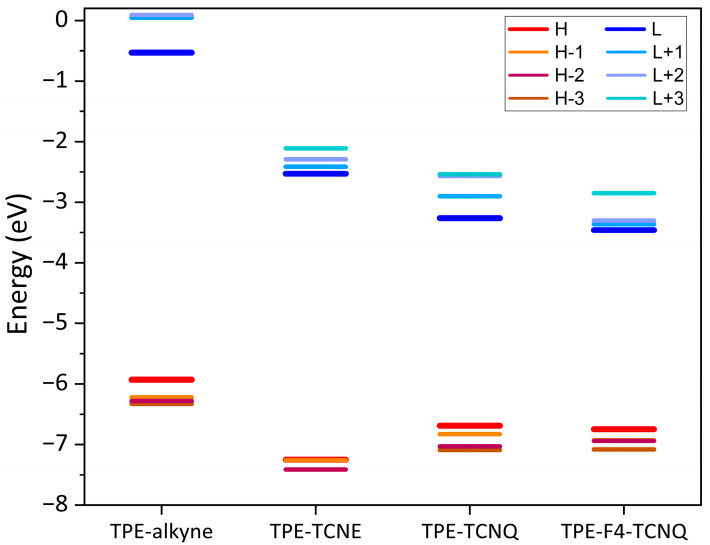
Energy level alignment of the four frontier occupied and virtual molecular orbitals for the investigated systems in THF, as calculated at the CAM-B3LYP/6-311G (d,p) level.

The relative energy level alignment of the frontier orbitals of the TPE adducts was estimated from the DFT calculations and are displayed in Figure 9 when accounting for the solvent effects (i.e., THF used for the optical absorption spectra) via the Polarizable Continuum Model (PCM). As measured in cyclic voltammetry, the calculated HOMO–LUMO gaps decrease when increasing the electron-withdrawing character of the adducts in the following order: F_4_-TCNQ > TCNQ > TCNE. It is worth remembering that the absolute values of the gap cannot be directly compared to the CV data since the calculations rely on Koopmans’ approximation (associating the ionization potential to the HOMO of the neutral molecule and the electron affinity to the LUMO energy). Moreover, the amplitude of the HOMO–LUMO gap critically depends on the choice of the DFT functional. Nevertheless, comparing the evolution of the HOMO and LUMO energies on a relative basis is meaningful. Doing so, we observe that the shift between the frontier energy levels of the various TPE adducts nicely matches the experimental trends (see Figure 9).

### 2.5. Excited-State Calculations

**Figure 10:**
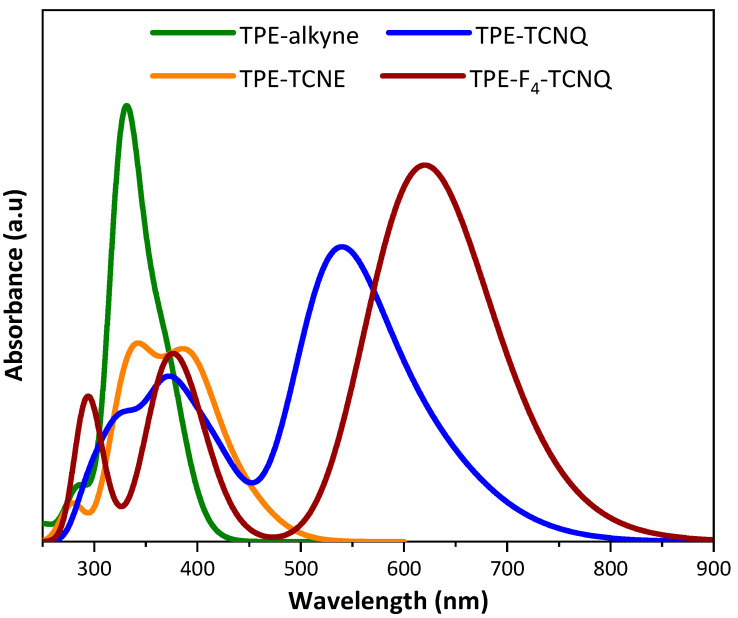
UV–Visible absorption spectra of the investigated compounds in THF simulated at the TD-DFT/CAM-B3LYP level.

The absorption spectra of the TPE compounds simulated at the TD-DFT level using the same functional and base set are shown in Figure 10 (see also Figure S47). The Gaussian broadening used to generate the UV–Visible absorption spectra is set to σ= 0.25 eV to match the width of the experimental bands. The computed electronic transitions, as well as their corresponding oscillator strength and assignment, are listed in Table S1, Supplementary Materials.

**Figure 11:**
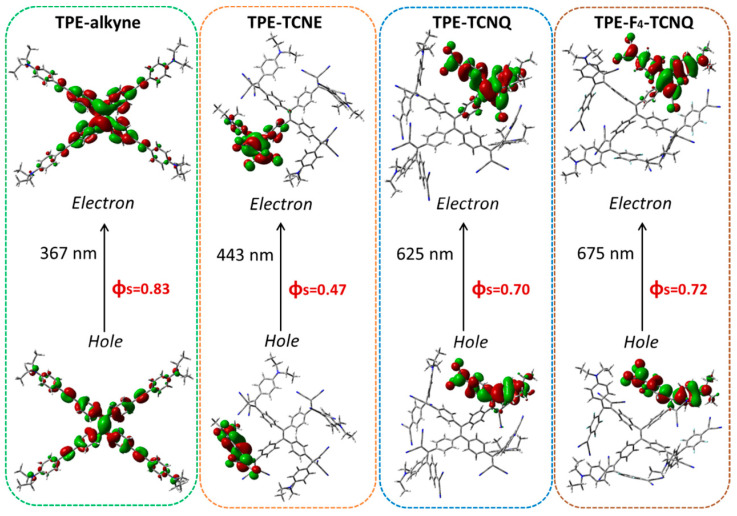
The hole and electron Natural Transition Orbitals (NTOs) for the lowest excitation of the investigated compounds with the associated energy, and φ_s_ descriptor, computed at the CAM-B3LYP/6-311G(d,p). Isovalues are set to 0.02 e/au^3^.

For TPE-alkyne, the lowest excitation occurs at 367 nm, which is in very good agreement with the strong absorption band experimentally observed in its UV−Visible absorption spectrum (λ_max_ = 359 nm, Table 1, Figure 3). This corresponds to a transition from the HOMO to the LUMO level (see Table S1, Supplementary Materials). Conversely, the absorption spectra of the TPE adducts exhibit an additional absorption band with a CT character in the visible–near infrared region. For TPE-TCNE, TPE-TCNQ and TPE-F_4_-TCNQ, the lowest excitation occurs at 443 nm, 625 nm and 675 nm, respectively. To further analyze the nature of these excitations, we performed an NTO analysis that describes globally for each given excitation where the electron is excited from and where it is promoted during the excitation, i.e., as a single-particle transition from an effective occupied to virtual orbital [97]. We refer to the occupied and virtual NTOs as “hole” and “electron” transition orbitals, respectively. Note that the NTOs are not the same as the occupied and virtual molecular orbitals calculated in the ground state. Moreover, we have also calculated the spatial overlap φs between the electron and hole density in order to quantitatively evaluate the charge-transfer character of an electronic transition; φs = 1 for a purely localized character and φs = 0 for a pure charge transfer excitation [98,99]. In the case of TPE-TCNE, the lowest excitation at 443 nm is predominantly assigned to a transition from a hole orbital localized on the extremity of a TCBD arm to an electron orbital localized on the inner part of the same TCBD arm and including a part of the TPE core. This graphical picture of NTOs is consistent with the moderate spatial overlap φs value of 0.47 calculated for this transition (see Figure 11). Moving to TPE-TCNQ and TPE-F_4_-TCNQ, the dominant transition contributing to the lowest excitation at 625 nm and 675 nm, exhibits both a weaker charge-transfer character compared to TPE-TCNE due to the strong spatial overlap between the hole and electron NTO orbitals as evidenced by a larger φs index (0.70 and 0.72, respectively) (see Figure 11). For all TPE adducts, the high oscillator strength associated with the lowest excitation is attributed to this strong intra-branch character. Interestingly, the simulated absorption spectra nicely reproduce the experimental bathochromic shift of the CT absorption band. Going from TPE-TCNE, TPE-TCNQ to TPE-F_4_-TCNQ, a systematic red shift in the CT absorption band as well as an increase in its intensity are noticed, which is fully in line with the increase in the electron-withdrawing strength of the TPE adducts and the decrease in the CT character, respectively. This red shift can be evaluated by computing the energy difference between the main peaks of the lowest charge transfer band of the TPE conducts. Thus, moving from TPE-TCNE (E_max_ = 3.11 eV) to TPE-TCNQ (E_max_ = 2.27 eV), a red shift of 0.84 eV is computed whereas a red shift of 0.34 eV is obtained going from TPE-TCNQ (E_max_ = 2.27 eV) to TPE-F_4_-TCNQ (E_max_ = 1.93 eV) (see Figure S46). These results are in excellent agreement with experiments showing the same red shifts of 0.84 eV and 0.34 eV, respectively (see Table 1).

### 2.6. Photothermal Properties and Thermal Stability

**Figure 12:**
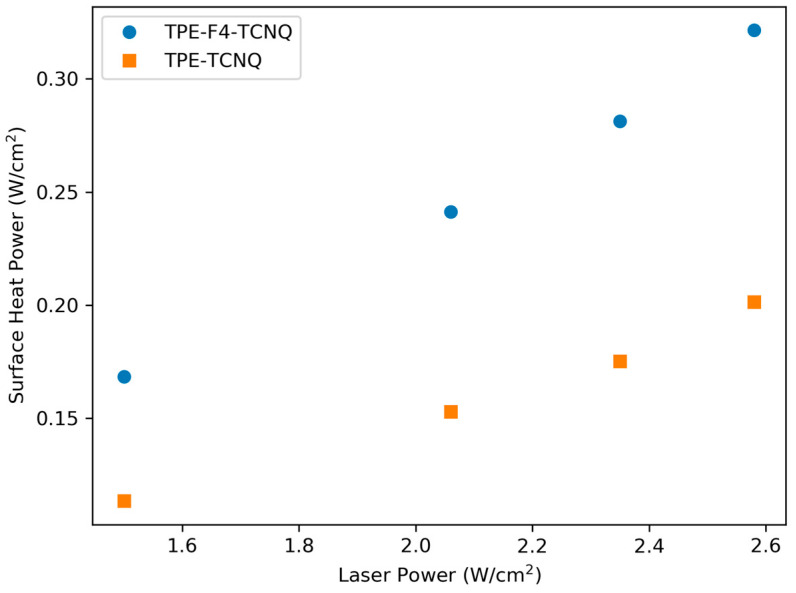
Comparison of the surface heat power vs. laser power at 808 nm for **TPE-TCNQ** (orange) and **TPE-F_4_-TCNQ** (blue) as thin films.

**Figure 13:**
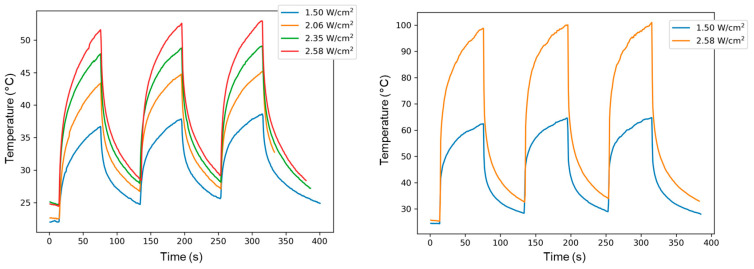
Photothermal conversion behavior of **TPE-F_4_-TCNQ** under 808 nm laser irradiation at different laser powers (1.50, 2.06, 2.35, and 2.58 cm^−2^), as a thin film (**left**) and as a powder (**right**).

Due to their high absorption in the red-NIR region, TPE-TCNQ and TPE-F_4_-TCNQ appear as suitable photothermal agents, relaxing to their ground state non-radiatively by the creation of phonons (vibrations) and thus affording an increase in the temperature [100]. To investigate the photothermal behavior of TPE-TCNQ and TPE-F_4_-TCNQ, the temperature changes of thin films and powders under a laser beam were measured. Figure S23 shows absorption spectra for TPE-TCNE, TPE-TCNQ and TPE-F_4_-TCNQ thin films. A wavelength of 808 nm was selected for this study for two reasons: (i) this wavelength is in the first biological transparency window (NIR-I, 750–1000 nm), (ii) this wavelength is located in a good absorbing region of the UV–Visible absorption spectra of both molecules. In similar conditions, TPE-F_4_-TCNQ shows a stronger photothermal conversion compared to TPE-TCNQ (Figure 12). Figure 13 displays photothermal conversion cycles for TPE-F_4_-TCNQ at different laser powers. The thermal cycles are reproducible, indicating that the molecules do not degrade under irradiation (see Figure S40). The first cycle was fitted using the COMSOL software to extract the surface heat power produced by the beam light irradiation. In the case of thin films and at the maximum power of the laser (2.58 W/cm^2^), the temperature rose to a maximum of 50 °C in less than one minute. If the thin film is replaced by a dense powder of TPE-F_4_-TCNQ, the temperature rises at almost 100 °C in less than one minute (Figure 13). This difference is explained by an increase in the amount of matter of the sample leading to an increase in light absorption in the case of the irradiation of the powder. Finally, to check the thermal stability of TPE-TCNQ and TPE-F_4_-TCNQ, a thermogravimetric analysis (TGA) under nitrogen flow was performed indicating no weight loss greater than 2% up to 320 °C (see Figure S36).

### 2.7. Concluding Remarks

Generally speaking, it is interesting to note that almost all the CA-RE derivatives described in the literature to date have spectral characteristics extremely close to those described in this paper when the electron donating group is a dialkyl or a diphenyl aniline [54,57,59,62,69,70,71,72,73,74,76,83,84,85,86,101,102,103,104,105,106,107,108,109,110,111,112,113,114,115,116,117]. In all cases, the ICT bands are found in the same regions within a few nm. The CT band A is between 450 and 470 nm and the CT band B between 680 and 710 nm for TCNQ and between 830 and 860 nm for F_4_-TCNQ. This indicates that whatever the starting alkyne, the charge transfers will only involve the cyanated parts and the aniline. This supposition is well supported by the TD-DFT calculations that showed that the central core played a very negligible role in the frontier orbitals and thus in the electronic properties. The observed behavior is exclusively governed by the clicked moieties.

## 3. Methods and Materials

### 3.1. Materials

Dry tetrahydrofuran (THF), dichloromethane (DCM) and diethyl ether (Et_2_O) were obtained by using a solvent purification system Puresolve MD5 from Inert. Anhydrous N,N-dimethylformamide (DMF) (Acros, 99.8%), dichlorobenzene (TCI, 99%), anhydrous acetonitrile (Alfa Aesar, 99.8%) and absolute ethanol (VWR) were used as received. Triphenylphosphine (Fluorochem, 99%), *n*-tetrabutylammonium fluoride (Sigma-Aldrich, 1M in THF), 1,1,2,2-tetraphenylethene (TPE) (Fluorochem, 95%), bromine (Sigma-Aldrich, 99%), copper iodide (Alfa Aesar, 99.998%), 4-ethynyl-N,N-dimethylaniline (Aldrich, 97%), diisopropylamine (Aldrich, 99.5%), 4-iodoaniline (Alfa Aesar, 98%), 1-bromododecane (ABCR, 99%), trimethylsilylacetylene (Fluorochem, 99%), 4,4′-dibromobenzophenone (Fluorochem, 98%), titanium (IV) chloride (Merck, 97%), zinc dust (Aldrich, 98%), pyridine (Aldrich, 99.8%), tetracyanoethylene (TCNE) (Aldrich, 96%), 7,7,8,8-Tetracyanoquinodimethane (TCNQ) (Alfa Aesar, 98%), 2,3,5,6-Tetrafluoro-7,7,8,8-tetracyanoquinodimethane (F_4_-TCNQ) (Fluorochem, 95%) were used as received. Tetrakis(triphenylphosphine)-palladium (0) [Pd(PPh_3_)_4_], was prepared according to literature procedures [113]. Ph-I-N-C_12_, Ph-SiMe_3_-N-C_12_ and Ph-alkyne were synthesized according to procedures described in the literature [114]. Reactions were monitored by thin-layer chromatography (TLC) using Merck© TLC Silica gel 60 F254 plates. Flash chromatography was carried out using a Biotage^®^ Isolera™ System (UV-Vis 200 nm–800 nm detector) over silica cartridges (SNAP Ultra or Sfär HC D).

### 3.2. Characterization Methods

Unless otherwise stated, measurements were done on the equipment available at the Plateforme d’Analyse et Caractérisation (PAC) of the Balard Institute, CNRS, University of Montpellier, France.

IR spectra were carried out on a Perkin Elmer Spectrum 2 FT-IR instrument using the attenuated total reflectance (ATR) measurement mode (diamond crystal). The wavenumber range analyzed was 500–4000 cm^−1^ and the optical resolution of the instrument was 4 cm^−1^.

^1^H NMR spectra were recorded at 273 K or 298 K on a Bruker Avance III 400 MHz NMR spectrometer using a BBFO probe or a Bruker Avance III 500 MHz NMR spectrometer using a Prodigy BBO Z-gradient CryoProbe or a Bruker Avance III 600 MHz NMR spectrometer using a Prodigy TCI Z-gradient CryoProbe and calibrated to TMS on the basis of the relative chemical shift of the residual non-deuterated solvent as an internal standard (CD_2_Cl_2_: δ = 5.32 ppm, CDCl_3_: δ = 7.26 ppm, DMSO-d_6_: δ = 2.50 ppm, THF-d_8_: δ = 3.58 ppm). ^13^C{^1^H} NMR spectra were recorded at 298 K on a Bruker Avance III 500 MHz NMR spectrometer using a Prodigy BBO Z-gradient CryoProbe or a Bruker Avance III 600 MHz NMR spectrometer using a Prodigy TCI Z-gradient CryoProbe and calibrated to TMS on the basis of the relative chemical shift of the residual non-deuterated solvent as an internal standard (CD_2_Cl_2_: δ = 53.84 ppm, CDCl_3_: δ = 77.16 ppm, DMSO-d_6_: δ = 39.52 ppm, THF-d_8_: δ = 67.21 ppm). ^19^F NMR spectra were recorded at 298 K on a Bruker Avance III 400 MHz NMR spectrometer using a BBFO probe.

The MS (MALDI) spectra were recorded on a Rapiflex (Bruker) with Matrix-Assisted Laser Desorption Ionization (MALDI) source and a TOF analyzer. The mass spectra were recorded in positive mode between 500 and 15,000 Da in reflector mode for TPE-adduct. Samples were incorporated in a DCTB matrix. Mass accuracy measurement were done at UMONS, Organic Synthesis and Mass Spectrometry Laboratory (S2MOs, Mons, Belgium), by using a Waters QToF Premier mass spectrometer equipped with Matrix-Assisted Laser Desorption/Ionization source. A Nd-YAG laser of 355 nm with a maximum pulse energy of 65 μJ delivered to the sample at 50 Hz repeating rate is used. Time-of-flight mass analyses were performed in the reflection mode at a resolution of about 10 k (*m*/*z* 569). Trans-2-(3-(4-tert-butyl-phenyl)-2-methyl-2-propenylidene)malononitrile (DCTB) was used as the matrix and was prepared as a 40 mg/mL solution in chloroform. The matrix solution (1 μL) was applied to a stainless-steel target and air-dried. Samples were dissolved in chloroform to obtain 1 mg/mL solutions. 1 μL aliquots of these solutions were applied onto the target area (already bearing the matrix crystals) and air-dried. Exact mass was determined by using poly(ethylene glycol) as internal reference.

The UV-Visible absorption spectra were recorded on a JASCO V-650 spectrophotometer in 10 mm quartz cells (Hellma). The molar extinction coefficients (ε) were determined by the plot of absorbance vs. concentration by preparing solutions at different concentrations in THF. The concentration range was chosen to remain in the linear range of the Beer–Lambert relationship (A ca. 0.2–0.8).

The optical gap (Eg) was determined by the Tauc method [115]. The onset wavelength of the absorption spectra was determined by the intersection of the straight line fitted to the right-hand side of the maximum with the baseline of the absorption.

Thin film were realized by deposition of ca. 10^−5^ mol.l^−1^ THF solutions on a quartz plate followed by the evaporation of the solvent. The operation was repeated until an absorbance of ca. 0.25 was measured at the maximum of the Intramolecular Charge Transfer (ICT) band.

The emission spectra in powder and in solution for TPE-TCNE, TPE-TCNQ and TPE-F_4_-TCNQ adducts were recorded in the Laboratoire de Chimie de l’ENS Lyon recorded on a fluorescence spectrofluorimeter (Fluorolog-3, Horiba-Jobin Yvon) equipped with R2658 photomultiplier tube (Hamamatsu, water cooling) or Synapse InGaAs/Symphony II (Horiba, nitrogen cooling) as detectors. The steady-state luminescence was excited by non-polarized light from a 450 W xenon CW lamp.

The measurement of quantum yields were carried out with an FLS980 integrating sphere (Edinburgh Instruments) connected to a FS920 fluorescence spectrophotometer (Edinburgh Instruments), equipped with a calibrated photomultiplier in a Peltier (air cooled) housing (R928P, Hamamatsu) operating up to 900 nm, with a 450 W continuous xenon arc lamp as the excitation source.

Measurements were performed in the Institut des Sciences Chimiques de Rennes –France (ISCR). For TPE-adducts, CVs were carried out on a 10^−3^ mol.L^−1^ solution of sample in CH_2_Cl_2_/[NBu_4_][PF_6_] 0.1 mol.l^−1^. CVs were recorded on a Biologic SP-50 instrument at 0.1 V s^−1^ on a platinum\u0002 disk electrode. Potentials were measured versus a KCl saturated calomel electrode (SCE).

ESR spectra were recorded on a Bruker Elexsys E500 CW continuous wave, X band (9.8 GHz) spectrometer equipped with ER4122 SHQ cavity at room temperature. Sample was diluted in THF and introduced in a quartz tube. The window was 10 mT (100 G) centered around g = 2 with 2 G of amplitude modulation, 100 kHz of frequency modulation, 10 dB of micro-wave power and 70 dB of gain.

Thermogravimetric analyses (TGA) were carried out on a STA 449 F1 Jupiter Netzsch analyzer under dry nitrogen at a heating rate of 10 °C.min^−1^.

Photothermal measurements were realized on samples of TPE-TCNE and TPE-F_4_-TCNQ. Two different deposits were realized with both samples. First bulk materials (powders) were placed on a quartz surface. Secondly, thin films of both samples were deposited on quartz slide from diluted THF solutions (*ca.* 1.10^−4^ mol.L^−1^). Then, we used a 808 nm laser (Kamax society, Limoges, France) with an adjustable power from 0 to 2.58 W/cm^2^. The laser spot surface was 0.32 cm^2^. The temperature of the surface was measured using an OPTRIS PI 450 thermal camera (Media Mesures, Bouc Bel Air, France). The samples were deposited on a polystyrene surface in contact with air to limit heat transfer with the support.

Photothermal simulations were realized using the COMSOL software [116]. The heat equation was solved simultaneously with the simplified Navier–Stokes equation to take into account the heat propagation and the convection terms present for the air. The model consisted of a quartz substrate, with a surface heat source to simulate the heat produced by the laser absorption of the TPE-TCNQ or TPE-F_4_-TCNQ sample layer, deposited on a polystyrene support.

### 3.3. Calculations Details

The ground-state geometry of the molecules has been first optimized without any symmetry constraint in the gas phase and the electronic properties of the optimized structures have been next determined in gas phase, in THF and in DCM used in the CV measurements. The vibrational frequencies associated to the optimized structures were also calculated to verify that they correspond to local minima on the energy surface. The absorption spectra and the vertical electronic transitions have been next computed at the TD-DFT level and compared to the corresponding experimental data. All calculations have been carried out using the long-range hybrid Cam-B3LYP functional [117] and a 6-311G (d,p) basis set within Revision D.01 of the Gaussian 09 program package [5]. The solvent effects in THF, used for the spectroscopic measurements, were simulated by means of the polarizable continuum model (PCM) [118].

### 3.4. Synthesis

**Bz-alkyne**. 4,4′-dibromobenzophenone (0.90 g, 2.66 mmol) was introduced into a 100 mL two neck round-bottom flask under argon atmosphere with 30 mL of anhydrous THF. Copper (I) iodide (20 mg, 0.11 mmol, 4 mol%), bis(triphenylphosphine)palladium (II) chloride (77 mg, 0.11 mmol, 4 mol%) and triphenylphosphine (13 mg, 0.05 mmol, 2 mol%) were then added (solution 1). In parallel, Ph-alkyne (2.90 g, 6.38 mmol, 2.4 eq) was introduced into a Schlenk tube under argon atmosphere with 10 mL of anhydrous THF and 20 mL of diisopropylamine (solution 2). Once prepared, solution 2 was transferred into solution 1 through cannula. The mixture was then heated to 65 °C overnight. Once judged complete, the solvent was removed under reduced pressure. The crude mixture was first filtrated on a silica gel column chromatography with cyclohexane before being purified by column chromatography on silica gel using gradient elution (100% cyclohexane to 70% cyclohexane / 30% dichloromethane). The fraction corresponding to the product was concentrated and then, diluted with a mixture of dichloromethane (5 mL) and acetone (50 mL). The solution was cooled. The resulting precipitate was filtered leading to Bz-alkyne as a yellow-orange powder. Yield 73% (2.10 g, 1.93 mmol). FTIR-ATR (cm^−1^): 2202 (νC≡C), 1651 (νC=O). ^1^H NMR (500 MHz, CD_2_Cl_2_, δ): 7.75 (d, 4H, ^3^*J*_H-H_ = 8.5 Hz), 7.58 (d, 4H, ^3^*J*_H-H_ = 8.5 Hz), 7.37 (d, 4H, ^3^*J*_H-H_ = 9.0 Hz), 6.60 (d, 4H, ^3^*J*_H-H_ = 9.0 Hz), 3.29 (t, 8H, ^3^*J*_H-H_ = 7.7 Hz), 1.63–1.55 (m, 8H), 1.37–1.21 (m, 72H), 0.88 (t, 12H, ^3^*J*_H-H_ = 7.0 Hz) ppm. ^13^C{^1^H} NMR (126 MHz, CD_2_Cl_2_, δ): 195.3, 148.9, 136.3, 133.5, 131.2, 130.5, 129.2, 111.6, 108.0, 95.2, 87.2, 51.4, 32.5, 30.2, 30.2, 30.1, 30.0, 29.9, 27.6, 27.6, 23.2, 14.4 ppm. HRMS (Maldi, DCTB): *m*/*z* [M]^●+^ calc for [C_77_H_116_N_2_O]^●+^: 1084.9088; found: 1084.9081 (δ = 0.6 ppm).

**TPE-alkyne**. Bz-alkyne (600 mg, 0.55 mmol) and zinc dust (111 mg, 1.70 mmol, 3 eq) were dissolved in anhydrous THF (30 mL) into a 100 mL three neck round bottom flask under argon atmosphere. Titanium (IV) chloride (120 µL, 1.11 mmol, 2 eq) was added dropwise at −78 °C. After return at room temperature, pyridine (20 µL, 0.44 mmol, 0.4 eq/TiCl_4_) was added and the solution was stirred during 30 min at room temperature. The mixture was then heated at 65 °C overnight. The progress of the reaction was followed by TLC using *n*-hexane 96%/4% dichloromethane as eluent. When judged complete, the mixture was cooled at room temperature and then, quenched with of a 10% aqueous solution of K_2_CO_3_ (60 mL). The aqueous layer was extracted with dichloromethane (5 × 50 mL). The organic layer was then dried over anhydrous MgSO_4_ and the solvent was removed under reduced pressure. The crude product was purified by column chromatography on silica gel using isocratic elution (90% *n*-hexane/9% dichloromethane/1% triethylamine). The product was isolated as an orange oil. Yield: 312 mg (0.15 mmol, 53%). FTIR-ATR (cm^−1^): 2208 (νC≡C). ^1^H NMR (500 MHz, CDCl_3_, δ): 7.33 (d, 8H, ^3^*J*_H-H_ = 8.4 Hz), 7.25 (d, 8H, ^3^*J*_H-H_ = 8.4 Hz), 6.98 (d, 8H, ^3^*J*_H-H_ = 7.8 Hz), 6.55 (d, 8H, ^3^*J*_H-H_ = 7.8 Hz), 3.25 (t, 16H, ^3^*J*_H-H_ = 7.4 Hz), 1.62–1.54 (m, 16H), 1.36-1.22 (m, 160H), 1.36–1.22 (t, 24H, ^3^*J*_H-H_ = 7.0 Hz) ppm. ^13^C{^1^H} NMR (126 MHz, CDCl_3_, δ): 148.0, 142.6, 140.8, 133.0, 131.6, 130.9, 122.7, 111.3, 108.9, 91.6, 87.3, 51.1, 32,1, 29.8, 29.8, 29.7, 29.5, 27.4, 27.3, 22.9, 14.3 ppm. HRMS (Maldi, DCTB) m/z: [M]^●+^ calc for [C_154_H_232_N_4_]^●+^: 2137.8277; found: 2137.8262 (δ = −0.7 ppm). UV-Vis (THF): λ (ε, L.mol^−1^.cm^−1^) = 359 (143 400), 294 (82 400) nm.

**TPE-TCNE**. TPE-alkyne (74 mg, 35.0 µmol) was introduced into a Schlenk tube under argon atmosphere. 10 mL of anhydrous dichloromethane as well as tetracyanoethylene (TCNE, 27 mg, 0.21 mmol, 6 eq) were added. The solution was stirred at room temperature during 1 h. The progress of the reaction was followed by TLC using *n*-hexane/CH_2_Cl_2_ (90/10 *v*/*v*) as eluent. When judged complete, the solvent was removed under vacuum. The product was then purified by column chromatography on silica gel using a gradient elution (90% *n*-hexane/10% dichloromethane to 80% *n*-hexane/20% dichloromethane). TPE-TCNE was isolated as a dark red film-forming solid. Yield: 65 mg (24 µmol, 70%). FTIR-ATR (cm^−1^): 2213 (νC≡N). ^1^H NMR (500 MHz, CDCl_3_, δ): 7.76 (d, 8H, ^3^*J*_H-H_ = 9.0 Hz), 7.54 (d, 8H, ^3^*J*_H-H_ = 8.5 Hz), 7.14 (d, 8H, ^3^*J*_H-H_ = 8.5 Hz), 6.70 (d, 8H, ^3^*J*_H-H_ = 9.0 Hz), 3.40 (t, 16H, ^3^*J*_H-H_ = 7.6 Hz), 1.64 (q, 16H, ^3^*J*_H-H_ = 6.2 Hz), 1.38–1.22 (m, 144H), 0.88 (t, 24H, ^3^*J*_H-H_ = 6.8 Hz). ^13^C{^1^H} NMR (126 MHz, CDCl_3_, δ): 168.8, 162.0, 153.5, 146.6, 142.8, 133.1, 132.3, 132.0, 129.9, 117.6, 114.9, 114.1, 112.6, 112.2, 111.3, 87.7, 51.7, 32.1, 29.8, 29.7, 29.6, 29.5, 27.5, 27.2, 22.8, 14.3 ppm. HRMS (Maldi, DCTB) *m*/*z*: [M]^●+^ calc for [C_178_H_232_N_20_]^●+^: 2649,8769; found: 2649.8757 (δ = −0.5 ppm). UV-Vis (THF): λ (ε, L.mol^−1^.cm^−1^) = 469 (113 400), 307 (45 600) nm. Mp: 124–127 °C.

**TPE-TCNQ**. TPE-alkyne (100 mg, 47.0 µmol, 1 eq) was introduced into a Schlenk tube under argon atmosphere with dichlorobenzene (10 mL). 7,7,8,8-tetracyano-*p*-quinodimethane (TCNQ, 57.6 mg, 0.28 mmol, 6 eq) was added. The solution was stirred at 100°C during 1 h. The progress of the reaction was followed by TLC using *n*-hexane/CH_2_Cl_2_ (90/10 *v*/*v*) as eluent. The solvent was removed under vacuum. The residue was purified by column chromatography on silica gel with a gradient elution (90% *n*-hexane/10% dichloromethane to 80% *n*-hexane/20% dichloromethane). TPE-TCNQ was isolated as a dark film-forming solid. Yield: 66 mg (22.0 µmol, 47%). FTIR-ATR (cm^−1^): 2201 (νC≡N). ^1^H NMR (600 MHz, 273K, CD_2_Cl_2_, δ): 7.46 (d, 4H, ^3^*J*_H-H_ = 8.0 Hz), 7.43 (d, 8H, ^3^*J*_H-H_ = 8.6 Hz), 7.28 (d, 8H, ^3^*J*_H-H_ = 9.1 Hz), 7.20 (d, 4H, ^3^*J*_H-H_ = 8.1 Hz), 7.04 (d, 12H, ^3^*J*_H-H_ = 8.6 Hz), 6.93–6.81 (m, 4H), 6.71 (d, 8H, ^3^*J*_H-H_ = 9.1 Hz), 3.38 (t, 16H, ^3^*J*_H-H_ = 7.7 Hz,), 1.65–1.60 (m, 16H), 1.38–1.18 (m, 144H), 0.88 (t, 24H, ^3^*J*_H-H_ = 6.9 Hz) ppm. ^13^C{^1^H} NMR (151 MHz, 273K, CD_2_Cl_2_, δ): 172.5, 154.1, 152.2, 152.1, 146.3, 142.7, 136.4, 135.7, 135.1, 132.3, 131.0, 129.8, 124.7, 124.0, 115.9, 115.8, 113.6, 112.9, 112.5, 88.8, 51.7, 32.3, 30.0, 30.0, 30.0, 29.8, 27.6, 27.2, 23.1, 14.4 ppm. HRMS (Maldi, DCTB) *m*/*z*: [M]^●+^ calc for [C_202_H_248_N_20_]^●+^: 2954.0021; found: 2953.9946 (δ = −2.5 ppm). UV-Vis (THF) λ (ε, L.mol^−1^.cm^−1^) = 688 (155 000), 468 (78 500), 337 (113 900) nm. Mp: 115–119 °C.

**TPE-F_4_-TCNQ**. TPE-alkyne (100 mg, 47.0 µmol) was introduced into a Schlenk tube under argon atmosphere with dichlorobenzene (10 mL). 2,3,5,6-Tetrafluoro-7,7,8,8-tetracyanoquinodimethane (F_4_-TCNQ, 77.9 mg, 0.28 mmol, 6 eq) was added. The solution was stirred at 100 °C during 1 h. The progress of the reaction was followed by TLC using 20% n-hexane/80% dichloromethane as eluent. The solvent was removed under vacuum. The product was purified by column chromatography on silica gel with a gradient elution (20% n-hexane/80% dichloromethane to 100% dichloromethane). TPE-F_4_TCNQ was isolated as a dark film-forming solid. Yield: 55 mg (22.0 µmol, 36%). FTIR-ATR (cm^−1^): 2196 (νC≡N). ^1^H NMR (400 MHz, CDCl_3_, δ): 7.51–7.29 (m, 16H), 7.15–6.99 (m, 8H), 6.90 (d, 8H, ^3^*J*_H-H_ = 8.1 Hz), 3.74–3.50 (m, 16H), 1.83–1.70 (m, 16H), 1,35–1.19 (m, 144H), 0.88 (t, 24H, ^3^*J*_H-H_ = 6.8 Hz) ppm. ^13^C{^1^H} NMR (126 MHz, CDCl_3_, δ): 172.0, 156.5, 146.6, 146.1, 145.6, 143.5, 142.8, 140.5, 138.8, 138.5, 134.4, 133.7, 132.0, 130.1, 117.5, 116.3, 113.3, 113.1, 112.1, 107.3, 89.4, 53.5, 53,4, 32.0, 29.8, 29.7, 29.7, 29.6, 29.4, 28.5, 27.1, 22.8, 14.3 ppm. ^19^F NMR (376 MHz, CDCl_3_, δ): −134.1, −134.4, −143.1, 143.4 ppm. HRMS (Maldi, DCTB) *m*/*z*: [M]^●+^ calc for [C_202_H_232_F_16_N_20_] ^●+^: 3241.8513; found: 3241.8586 (δ = 2.3 ppm). UV-Vis (THF) λ (ε, L.mol^−1^.cm^−1^) = 849 (163 400), 497 (73 600), 348 (97 500) nm. Mp: 160–164 °C.

## 4. Conclusions

In conclusion, we have successfully applied the [2 + 2] CA-RE click reactions to a TPE platform adequately functionalized with electron-rich alkynes to obtain three cycloadducts, namely TPE-TCNE, TPE-TCNQ and TPE-F_4_-TCNQ. All these cycloadducts were fully characterized by NMR spectroscopy and HR-MS spectrometry. As confirmed by ESR spectroscopy, TPE-F_4_-TCNQ showed a substantial paramagnetic behavior in the solid state. As reported elsewhere, all compounds exhibited strong absorption bands in the NIR region of their UV–Visible absorption spectra, whose positions depend on the electron-withdrawing character of the clicked moieties. The origin of these bands was attributed by TD-DFT calculations to intramolecular charge transfers within the clicked moieties, indicating that the TPE core played a negligible role in the frontier orbitals and thus in the electronic properties. As a result, the TPE-alkyne precursor was rather luminescent, with a QY of 21%, and displayed a marked AIE behavior, whereas, among the clicked compounds, only TPE-TCNE showed a very faint fluorescence accompanied by an AIE behavior. Because they are non-luminescent and their CT band is in the NIR domain, TPE-TCNQ and TPE-F_4_-TCNQ were found to be suitable candidates for photothermal studies. Upon irradiation with a laser beam at 808 nm, both compounds exhibited excellent photothermal properties; the best results being obtained with powders of TPE-F_4_-TCNQ. In this case, a temperature of 100 °C was reached within less than one minute without decomposition under a laser power of 2.58 W/cm². The present study shows that CA-RE click reactions provide an excellent strategy for conferring high performance photothermal properties to any molecular platform with appropriate functionalities and adequate thermal stability. This result is very interesting393 nce it suggests that every already published or future CA-RE derivatives of TCNQ or F_4_-TCNQ could be good candidates for photothermal applications.

## Figures and Tables

**Table 1:** Optical data (absorbance and fluorescence) for the investigated compounds recorded in solution or in the aggregated state.

	TPE-Alkyne	TPE-TCNE	TPE-TCNQ	TPE-F_4_-TCNQ
Absorbance in solution ^a^ (nm)/(ε (L.mol^−1^.cm^−1^))	294 (82,400) 359 (143,400)	307/(45,600) 469 (113,400)	337 (113,900) 468 (78,500) 688 (155,000)	348 (97,500) 497 (73,600) 849 (163,400)
Absorbance as thin films (nm)	-	476	471; 738	498; 864
λ_em max_ (solution ^a^/aggregated ^b^)	582/560/-	≈700/786	-	-
Φ_sol_ (%) ^a^	13%	-	-	-
Φ_aggr_ (%) ^b^	21%	ND	-	-
λ_onset_ (nm) ^c^	460	540	900	-
E_g opt_ (eV) ^d^	2.70	2.30	1.38	-

^a^ measured in THF, ^b^ 10% THF/90% H_2_O, ^c^ Estimated from the extrapolation of the absorption curve of TPE-TCNQ, ^d^ Calculated using the relation E = 1.6 × 10^−19^ (h.c/λ_onset_). ND: not determined (see Characterization Methods part).

**Table 2:** Electrochemical data extracted from cyclic voltammetry in dichloromethane with 0.2 M of NBu_4_PF_6_ as electrolyte (potentials versus saturate calomel electrode).

	TPE-Alcyne	TPE-TCNE	TPE-TCNQ	TPE-F_4_-TCNQ
E_1/2_ (V) ^a^	-	−0.45	−0.23	+0.09
		−0.88	−0.46	−0.21
E_p_^b^	+0.80	+1.31	+0.88	+0.97

^a^ E_1/2_ = (E_pc_ + E_pa_)/2, in which E_pc_ and E_pa_ correspond to the cathodic and anodic peak, respectively (this potential is calculated when the electron transfer is reversible) ^b^ E_p_ = potential peak (irreversible wave).

## Data Availability

Data is available in the Supplementary Material.

## Supplementary Materials

The following supporting information can be downloaded at: https://www.mdpi.com/article/10.3390/ijms24108715/s1.
