# Supplementary material for: Synthesis and Characterization of Tetraphenylethene AIEgen-Based Push–Pull Chromophores for Photothermal Applications: Could the Cycloaddition–Retroelectrocyclization Click Reaction Make Any Molecule Photothermally Active?
Source: Int J Mol Sci. 2023 May 13;24(10):8715. doi: 10.3390/ijms24108715 (PMC10218099; doi:10.3390/ijms24108715)
Supplement: Supplementary file 1 [file ijms-24-08715-s001.zip › ijms-2372434-supplementary.pdf]

Synthesis and Characterization of Tetraphenylethene AIEgen-Based Push-Pull Chromophores for Photothermal Applications: Could the Cycloaddition – Retroelectrocyclization Click Reaction Make any Molecule Photothermally Active?

Maxime Roger,[a] Yann Bretonnière,[b] Yann Trolez,[c] Antoine Vacher,[c] Imane Arbouch,[d] Jérôme Cornil,[d] Gautier Félix,[a] Julien De Winter,[e] Sébastien Richeter,[a] Sébastien Clément,*[,a] and Philippe Gerbier*[, a]

a) Univ. Montpellier, ICGM, UMR 5253, CNRS, ENSCM, 34293 Montpellier, France.
b) Univ. Lyon 1, ENSL, CNRS, UCBL, Laboratoire de Chimie UMR 5182, 69364 Lyon, France
c) Univ. Rennes, ISCR, UMR 6226, ENSCR, CNRS, 35000 Rennes, France.
d) Univ. Mons-UMONS, Laboratory for Chemistry of Novel Materials, 7000 Mons, Belgium.
e) Univ Mons-UMONS, Organic Synthesis and Mass Spectrometry Laboratory (S2MOs), 7000 Mons, Belgium.

Correspondence: sebastien.clement1@umontpllier.fr ; philippe.gerbier@umontpellier.fr

Table of content

I. IR, NMR and MS spectroscopies 6
1. **Bz-alkyne** 6
2. **TPE-alkyne** 9
3. **TPE-TCNE** 11
4. **TPE-TCNQ** 13
5. **TPE-F_4-TCNQ** 15
II. Absorption and fluorescence spectroscopies 18
III. Study of AIE behavior 18
IV. pH-dependent emission studies 19
V. Electrochemistry 20
1. **TPE-TCNE** 20
2. **TPE-TCNQ** 21
3. **TPE-F_4-TCNQ** 23
VI. Thermal and photothermal studies 24

VII. Calculations 27

Table of figures

Figure S1. FTIR-ATR spectrum of **Bz-alkyne.** 6

Figure S2: ^{1}H NMR spectrum (500 MHz, CD_2Cl_2, 298K) of **Bz-alkyne**. 6

Figure S3. ^{13} C{^{1}H} NMR spectrum (126 MHz, CD_2Cl_2, 298K) of **Bz-alkyne** 7

Figure S4. High resolution MALDI-TOF spectrum of **Bz-alkyne** (matrix: DCTB/NaI). 8

Figure S5. FTIR-ATR spectrum of **TPE-alkyne**. 9

Figure S6. ^{1}H NMR spectrum (400 MHz, $CDCl_3$, 298K) of **TPE-alkyne**. 9

Figure S7. ^{13}C{^{1}H} NMR spectrum (126 MHz, $CDCl_3$, 298K) of **TPE-alkyne**. 10

Figure S8. High Resolution MALDI-TOF mass spectrum of **TPE-alkyne** (matrix: DCTB/NaI). 10

Figure S9. FTIR-ATR spectrum of **TPE-TCNE**. 11

Figure S10. ^{1}H NMR spectrum (500 MHz, $CDCl_3$, 298K) of **TPE-TCNE**. 11

Figure S11. ^{13}C{^{1}H} NMR spectrum (126 MHz, $CDCl_3$, 298K) of **TPE-TCNE** 12

Figure S12. High resolution MALDI-TOF mass spectrum of **TPE-TCNE** (matrix: DCTB/NaI). 12

Figure S13. FTIR-ATR spectrum of **TPE-TCNQ**. 13

Figure S14. ^{1}H NMR spectrum (600 MHz, CD_2Cl_2, 273K) of **TPE-TCNQ**. 13

Figure S15. ^{13}C{^{1}H} NMR spectrum (151 MHz, CD_2Cl_2, 273K) of **TPE-TCNQ** 14

Figure S16. ^{1}H NMR spectrum (600 MHz, $CDCl_3$, blue, 4 and 3) and ^{1}H NMR spectrum (600 MHz, $CDCl_3$, red, 2 and 1) of **TPE-TCNQ** at 298 K and 278 K. 14

Figure S17. High resolution MALDI-TOF mass spectrum of **TPE-TCNQ** (matrix: DCTB/NaI). 15

Figure S18. FTIR-ATR spectrum of **TPE-F_4-TCNQ**. 15

Figure S19. ^{1}H NMR (400 MHz, $CDCl_3$, 298K) of **TPE-F_4-TCNQ**. 16

Figure S20. ^{13}C{^{1}H} NMR spectrum (126 MHz, $CDCl_3$, 298K) of **TPE-F_4-TCNQ**. 16

Figure S21. ^{19}F{^{1}H} NMR spectrum (376 MHz, $CDCl_3$, 298K) of **TPE- F_4-TCNQ** 17

Figure S22. High resolution MALDI-TOF mass spectrum of **TPE-F$_4$-TCNQ** (matrix: DCTB/NaI). 17

Figure S23. Absorption spectra of TPE adducts as thin films on quartz substrate. 18

Figure S24. iA) emission spectra ($\lambda_{exc.}$ = 360 nm) of **TPE-Alkyne** in various THF / water mixtures (C = 6.10^{-6} mol.L^{-1}). ; B) variation of the relative emission intensity vs. water fraction (I_{THF}: Intensity of the fluorescence of **TPE-alkyne** at the maximum emission in THF and I: Intensity of the fluorescence of TPE-alkyne at the maximum emission in various THF /water mixtures) and C) photographs of the corresponding mixtures under natural (left) and UV (left, 365 nm) light illumination. 18

Figure S25. A) emission spectra ($\lambda_{exc.}$ = 480 nm) of **TPE-TCNE** in various THF / water mixtures (C = 5.3.10^{-5} mol.L^{-1}) ; B) variation of the relative emission intensity vs. water fraction. 19

Figure S26. A) Reversible proposed acidification scheme for **TPE-alkyne** in THF/water solution ; B) variation of the emission spectra as a function of the pH (H^+ : addition of an aqueous HCl solution up to pH = 1 +/- 0.1 and OH^- : addition of an aqueous NaOH solution up to pH = 13 +/- 0.1) (C = 1.10^{-5} mol.L^{-1}, $\lambda_{exc.}$ = 360 nm) and C) drawing of the relative emission intensity variation upon pH cycling and corresponding images under natural and UV (365 nm) light illumination. 19

Figure S27. Cyclic voltammogram of **TPE-TCNE** in 0.2 M solution of Bu_4NPF_6 in CH_2Cl_2. (scan rate: 100 mV.s^{-1}; . electrode potential vs. SCE (saturated calomel electrode)). 20

Figure S28. Differential pulse voltammetry (DPV) of **TPE-TCNE** in 0.2 M solution of Bu_4NPF_6 in CH_2Cl_2 (scan rate: 100 mV.s^{-1}; . electrode potential vs. SCE).. 20

Figure S29. Differential absorption spectra obtained during the reduction of **TPE-TCNE** in 0.2 M solution of Bu_4NPF_6 in CH_2Cl_2 (scan rate: 100 mV.s^{-1}; . electrode potential vs. SCE). 21

Figure S30. Cyclic voltammogram of **TPE-TCNQ** in 0.2 M solution of Bu_4NPF_6 in CH_2Cl_2 (scan rate: 100 mV.s^{-1}; . electrode potential vs. SCE). 21

Figure S31. Differential pulse voltammetry (DPV) of **TPE-TCNQ** in 0.2 M solution of Bu_4NPF_6 in CH_2Cl_2 (scan rate: 100 mV.s^{-1}; . electrode potential vs. SCE). 22

Figure S32. Differential absorption spectra obtained during the reduction of **TPE-TCNQ** in 0.2 M solution of Bu_4NPF_6 in CH_2Cl_2. 22

Figure S33. Cyclic voltammogram of **TPE-F_4-TCNE** in 0.2 M solution of Bu_4NPF_6 in CH_2Cl_2 (scan rate: 100 mV.s^{-1}; . electrode potential vs. SCE). 23

Figure S34. Differential pulse voltammetry (DPV) of **TPE-F_4-TCNQ** in 0.2 M solution of Bu_4NPF_6 in CH_2Cl_2 (scan rate: 100 mV.s^{-1}; . electrode potential vs. SCE). 23

Figure S35. Differential absorption spectra obtained during the reduction of **TPE-F_4-TCNE** in 0.2 M solution of Bu_4NPF_6 in CH_2Cl_2. 24

Figure S36. Thermogravimetric analysis (TGA) of **TPE-TCNQ** (blue) and **TPE-F_4-TCNQ** (red) under N_2 flow. 24

Figure S37. Photothermal conversion behavior of **TPE-TCNQ** as a thin film under 808 nm laser irradiation at different laser powers (1.50, 2.06, 2.35, and 2.58 cm^{-2}) vs. Comsol fits. 25

Figure S38. Surface heat power vs. laser power at 808 nm for **TPE-TCNQ** as a thin film. 25

Figure S39. Photothermal conversion behavior of **TPE-TCNQ** as a powder under 808 nm laser irradiation at laser powers of 1.50 and 2.58 cm^{-2}. 25

Figure S40. Photothermal conversion behavior of **TPE-F_4-TCNQ** as a thin film under 808 nm laser irradiation at different laser powers (1.50, 2.06, 2.35, and 2.58 cm^{-2}) vs. Comsol fits. 26

Figure S41. Surface heat power vs. laser power at 808 nm for **TPE-TCNQ** as a thin film. 26

Figure S42. Photothermal conversion behavior of **TPE-F_4-TCNQ** as a powder under 808 nm laser irradiation at laser powers of 1.50 and 2.58 cm^{-2}. 26

Figure S43. Comparison of the surface heat power vs. laser power at 808 nm for **TPE-TCNQ** (orange) and **TPE-F_4-TCNQ** as thin films. 27

Figure S44. Optimized geometry of the investigated compounds computed at DFT-Cam-B3LYP/6-311G(d,p) theory level. 27

Figure S45. Energy alignment of the four frontier occupied and virtual molecular orbitals for the investigated systems in both gas phase and in THF/DCM solution, as calculated at CAM-B3LYP/6- 311G (d,p) level. 28

Figure S46. Shape of the four occupied and virtual molecular orbitals for **TPE-TCNE** in the gas phase. Energies are computed at the CAM-B3LYP/6-311G(d,p) level of theory in gas phase and in THF solution. Isovalues are set to 0.02 e/au^3. 28

Figure S47. Simulated UV-Vis absorption spectra of the investigated compounds in THF solution at the TDDFT/Cam-B3LYP level with all transition peaks. The main transition energy of the CT absorption band for TPE adducts is also pointed with an arrow.................. 30

Table of Tables

***Table S1**: Assignment of electronic excitations for the investigated compounds, as obtained at the TD-DFT/Cam-B3LYP level [a].* 28

I. IR, NMR and MS spectroscopies

1. Bz-alkyne

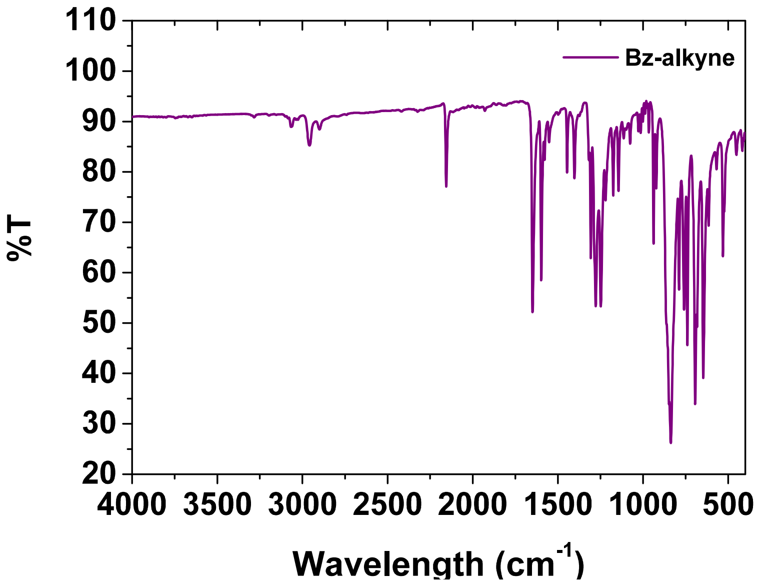

Figure S1. FTIR-ATR spectrum of **Bz-alkyne.**

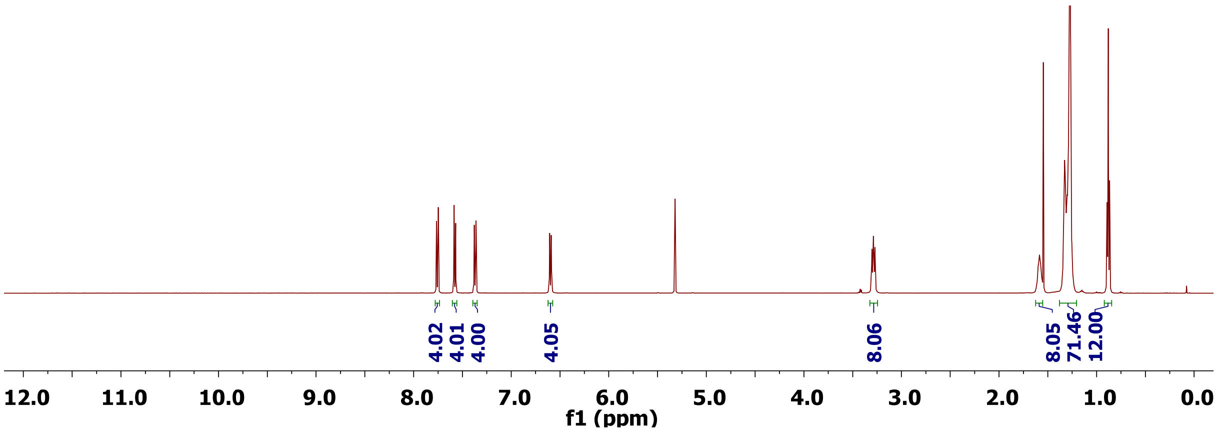

Figure S2. ^{1}H NMR spectrum (500 MHz, CD_2Cl_2, 298K) of **Bz-alkyne**.

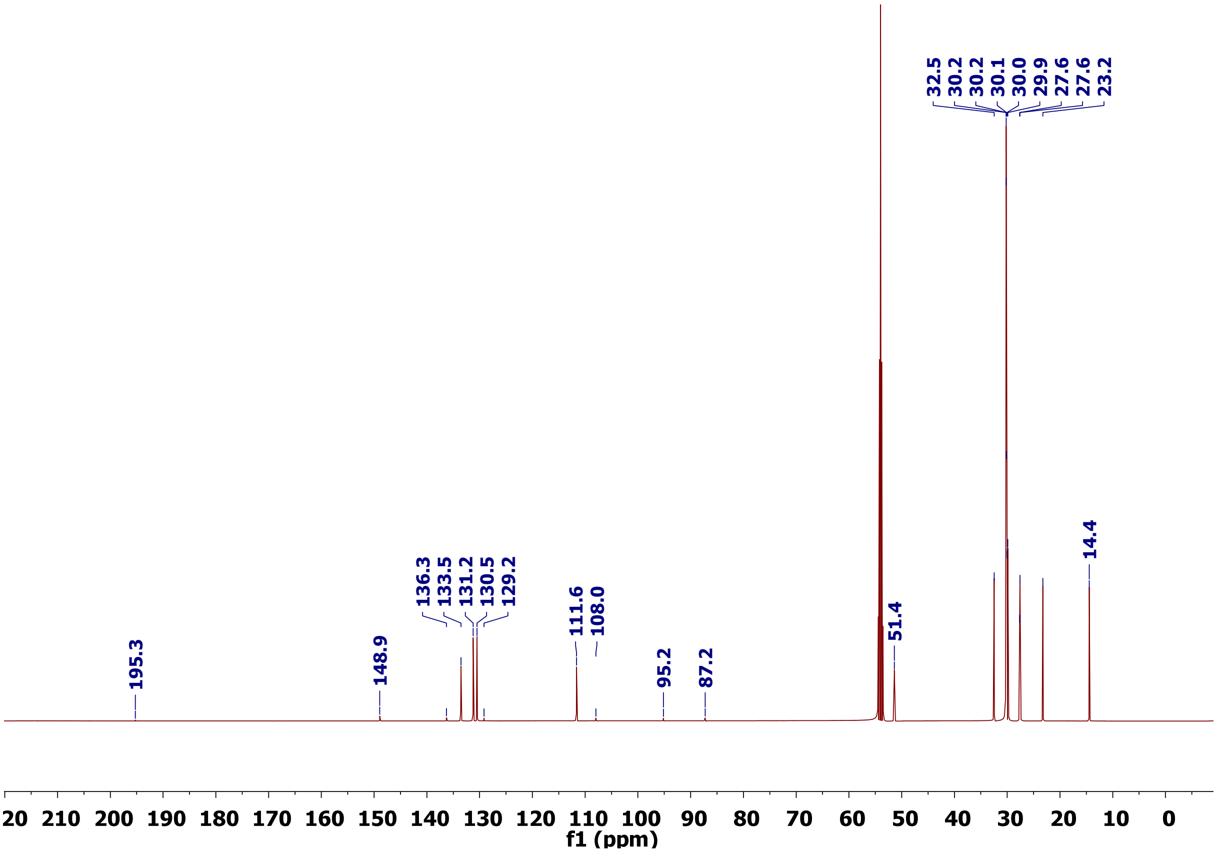

Figure S3. $^{13}C\{^{1}H\}$ NMR spectrum (126 MHz, CD_2Cl_2, 298K) of **Bz-alkyne**.

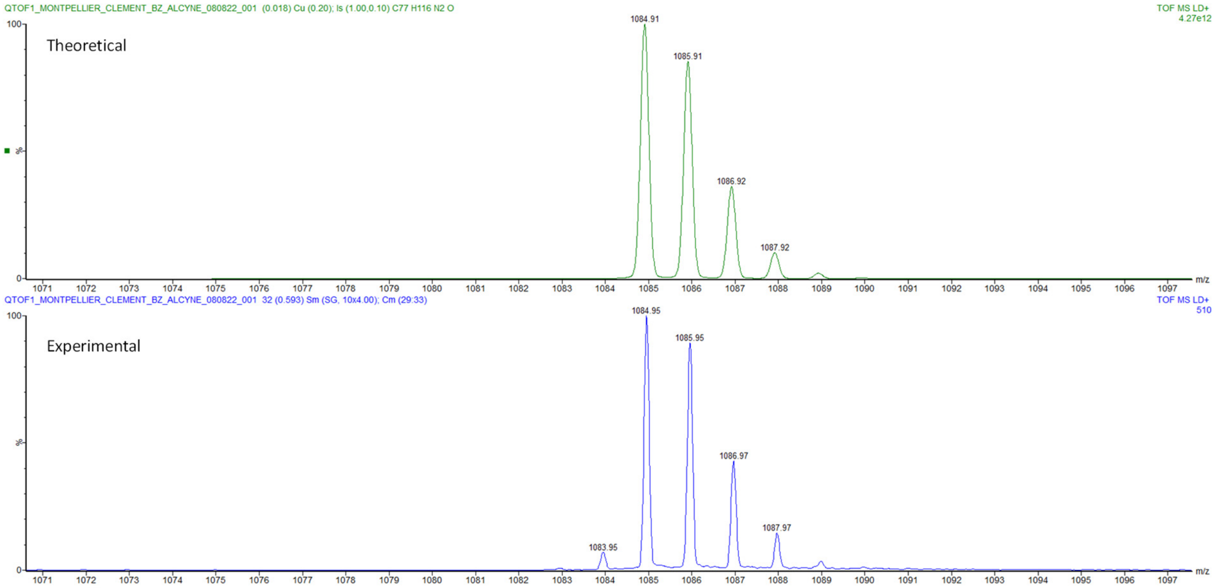

Elemental Composition Report

Single Mass Analysis
Tolerance = 25.0 PPM / DBE: min = -1.5, max = 200.0
Element prediction: Off
Number of isotope peaks used for i-FIT = 3

Monoisotopic Mass, Odd and Even Electron Ions
249 formula(e) evaluated with 9 results within limits (up to 50 closest results for each mass)
Elements Used:
C: 20-250 H: 0-250 N: 2-2 O: 0-20

Minimum:				-1.5				
Maximum:			5.0	25.0	200.0			
Mass	Calc. Mass	mDa	PPM	DBE	i-FIT	Norm	Conf(%)	Formula
1084.9081	1084.9088	-0.7	-0.6	21.0	87.6	0.088	91.53	C77 H116 N2 O
	1084.9053	2.8	2.6	-1.0	93.8	6.316	0.18	C59 H124 N2 O14
	1084.9146	-6.5	-6.0	12.0	91.3	3.754	2.34	C70 H120 N2 O6
	1084.8994	8.7	8.0	8.0	92.6	5.119	0.60	C66 H120 N2 O9
	1084.9205	-12.4	-11.4	3.0	93.8	6.324	0.18	C63 H124 N2 O11
	1084.9224	-14.3	-13.2	65.5	93.9	6.365	0.17	C64 H N2 O18
	1084.8935	14.6	13.5	17.0	90.9	3.419	3.27	C73 H116 N2 O4
	1084.9299	-21.8	-20.1	16.0	91.6	4.084	1.68	C74 H120 N2 O3
	1084.8841	24.0	22.1	4.0	95.5	8.040	0.03	C62 H120 N2 O12

Figure S4. High resolution MALDI-TOF spectrum of **Bz-alkyne** (matrix: DCTB/NaI).

2. TPE-alkyne

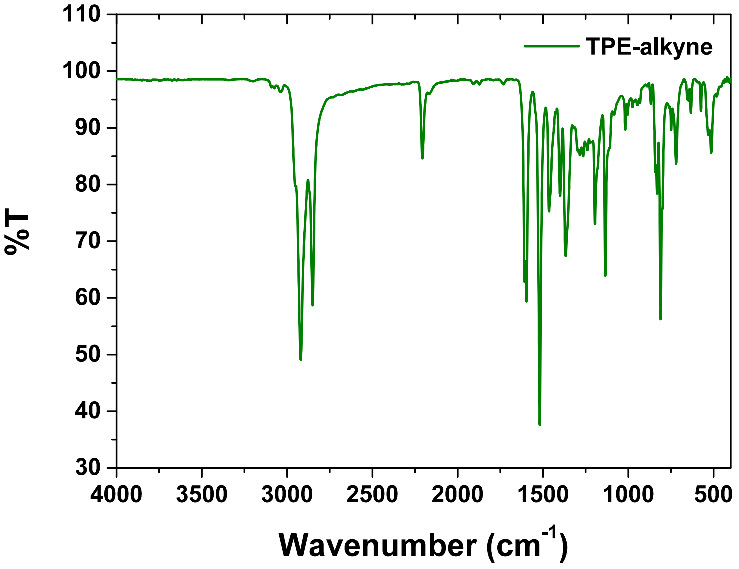

Figure S5. FTIR-ATR spectrum of **TPE-alkyne**.

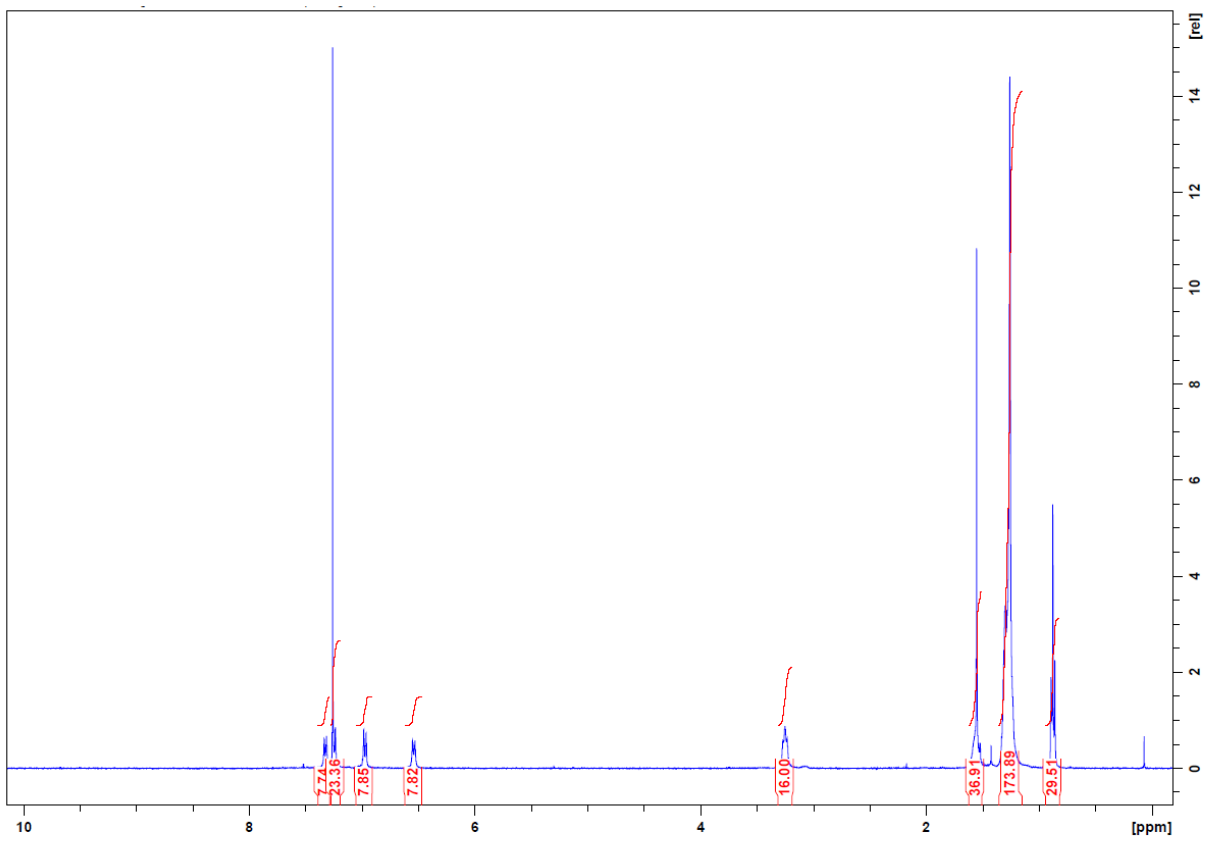

Figure S6. ^{1}H NMR spectrum (400 MHz, $CDCl_3$, 298K) of **TPE-alkyne**.

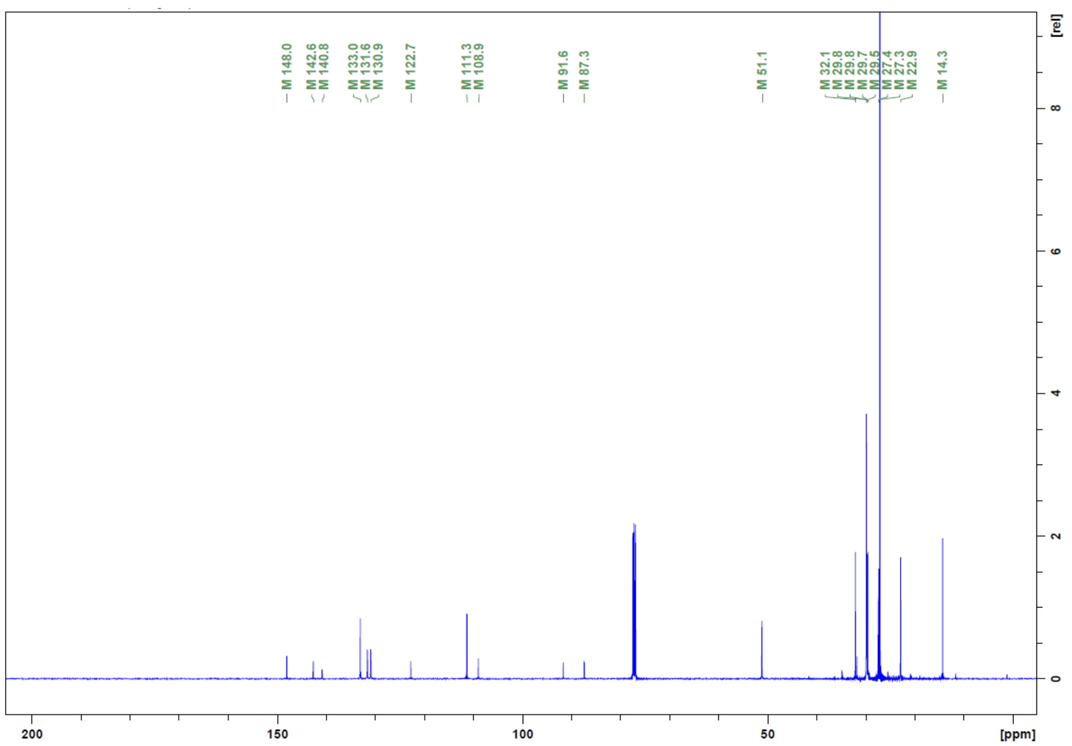

Figure S7. $^{13}C\{^{1}H\}$ NMR spectrum (126 MHz, $CDCl_3$, 298K) of **TPE-alkyne**.

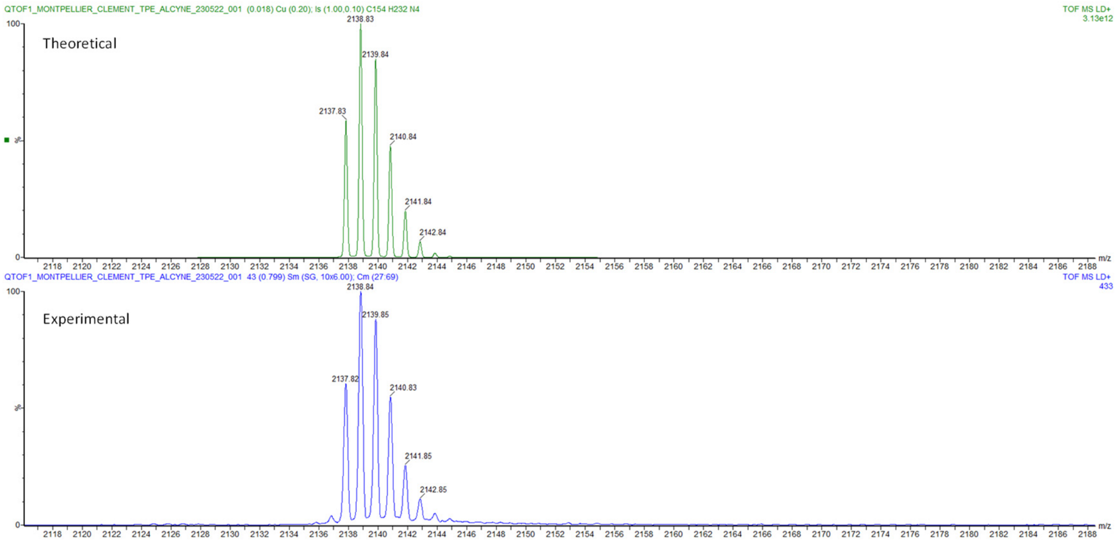

Elemental Composition Report

Single Mass Analysis
Tolerance = 25.0 PPM / DBE: min = -1.5, max = 150.0
Element prediction: Off
Number of isotope peaks used for i-FIT = 3

Monoisotopic Mass, Odd and Even Electron Ions
21 formula(e) evaluated with 2 results within limits (up to 50 closest results for each mass)
Elements Used:
C: 20-250 H: 0-250 N: 4-4

Minimum:				-1.5				
Maximum:			5.0	25.0	150.0			
Mass	Calc. Mass	mDa	PPM	DBE	i-FIT	Norm	Conf(%)	Formula
2137.8262	2137.8277	-1.5	-0.7	41.0	27.8	0.026	97.39	C154 H232 N4
	2137.8026	23.6	11.0	117.5	31.4	3.647	2.61	C165 H101 N4

Figure S8. High Resolution MALDI-TOF mass spectrum of **TPE-alkyne** (matrix: DCTB/NaI).

3. TPE-TCNE

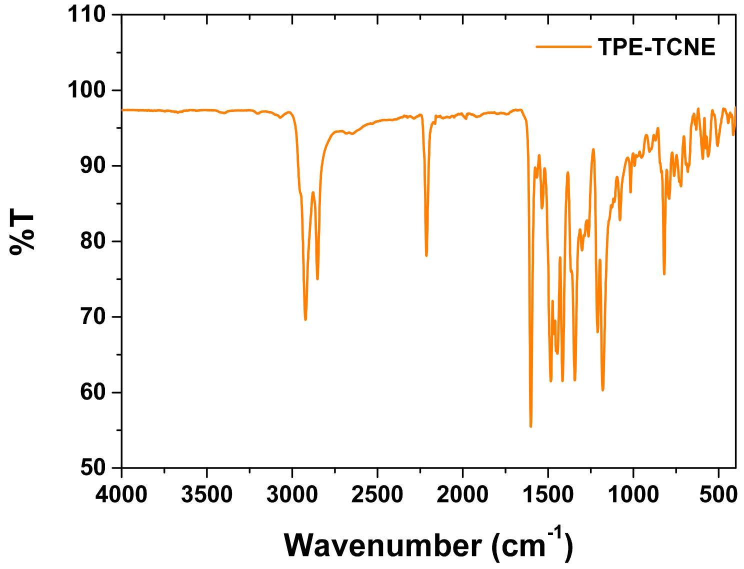

Figure S9. FTIR-ATR spectrum of **TPE-TCNE**.

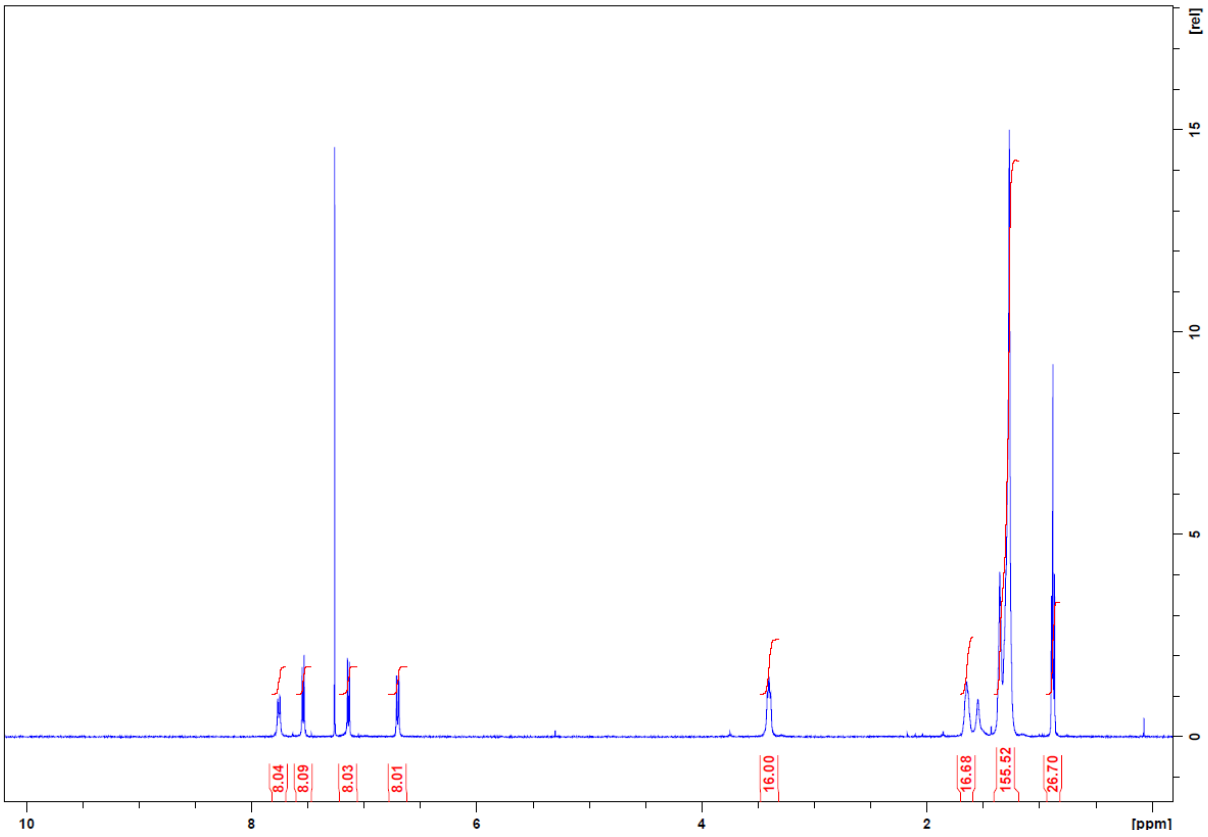

Figure S10. ^{1}H NMR spectrum (500 MHz, $CDCl_3$, 298K) of **TPE-TCNE**.

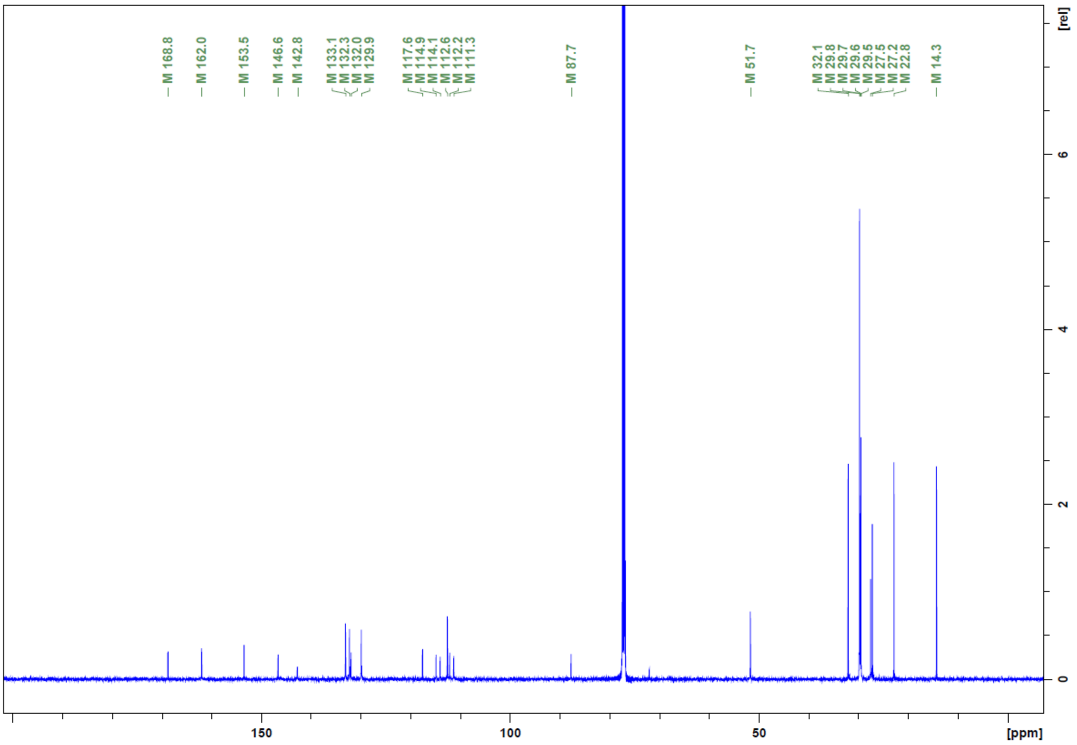

Figure S11. $^{13}C\{^{1}H\}$ NMR spectrum (126 MHz, $CDCl_3$, 298K) of **TPE-TCNE**.

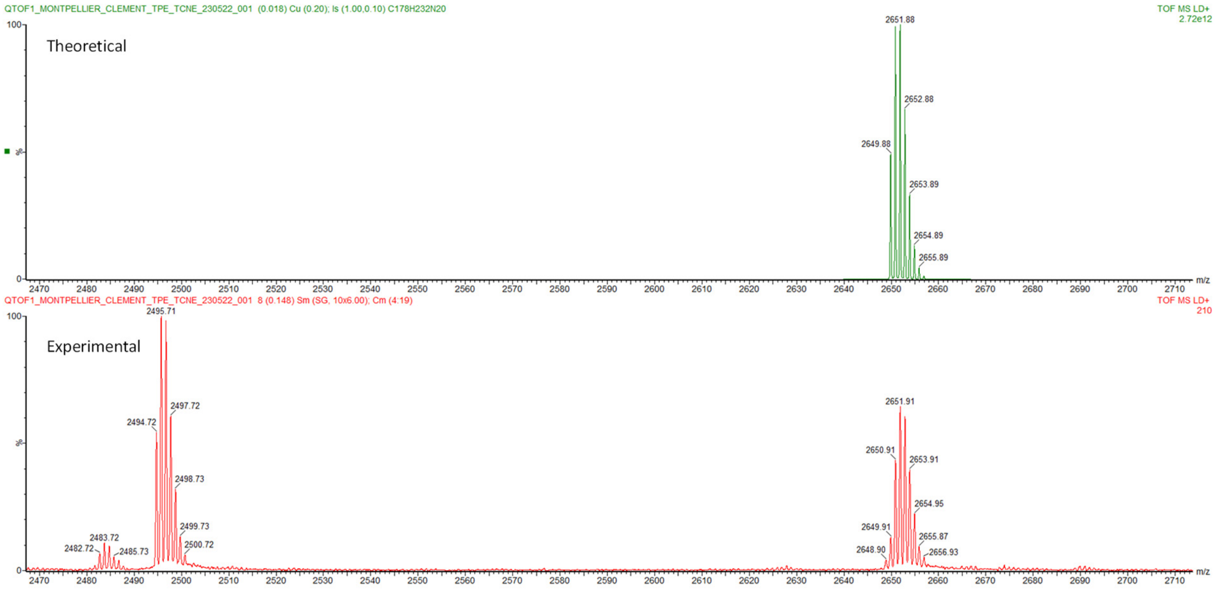

Elemental Composition Report

Single Mass Analysis
Tolerance = 25.0 PPM / DBE: min = -1.5, max = 200.0
Element prediction: Off
Number of isotope peaks used for i-FIT = 3

Monoisotopic Mass, Odd and Even Electron Ions
21 formula(e) evaluated with 2 results within limits (up to 50 closest results for each mass)
Elements Used:
C: 20-250 H: 0-250 N: 20-20

Minimum:				-1.5				
Maximum:			5.0	25.0	200.0			
Mass	Calc. Mass	mDa	PPM	DBE	i-FIT	Norm	Conf(%)	Formula
2649.8757	2649.8769	-1.2	-0.5	73.0	20.8	0.586	55.63	C178 H232 N20
	2649.8518	23.9	9.0	149.5	21.0	0.813	44.37	C189 H101 N20

Figure S12. High resolution MALDI-TOF mass spectrum of **TPE-TCNE** (matrix: DCTB/NaI).

4. TPE-TCNQ

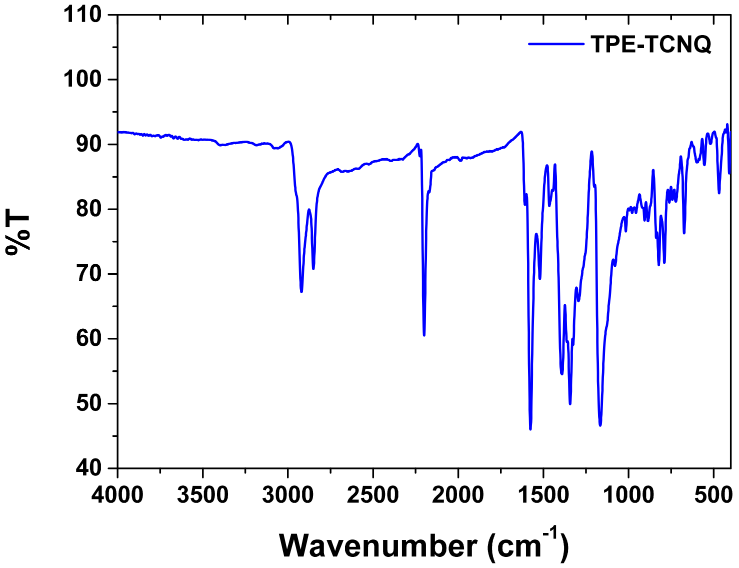

Figure S13. FTIR-ATR spectrum of **TPE-TCNQ**.

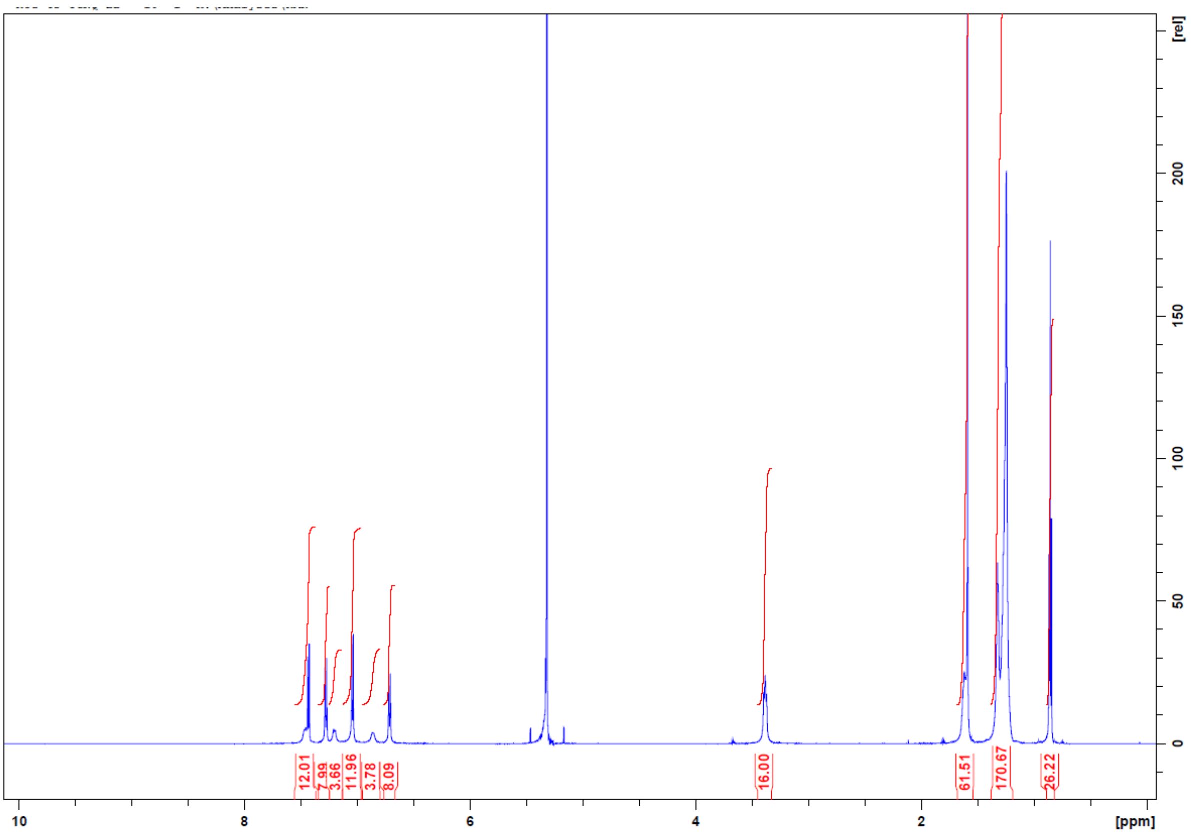

Figure S14. ^{1}H NMR spectrum (600 MHz, CD_2Cl_2, 273K) of **TPE-TCNQ**.

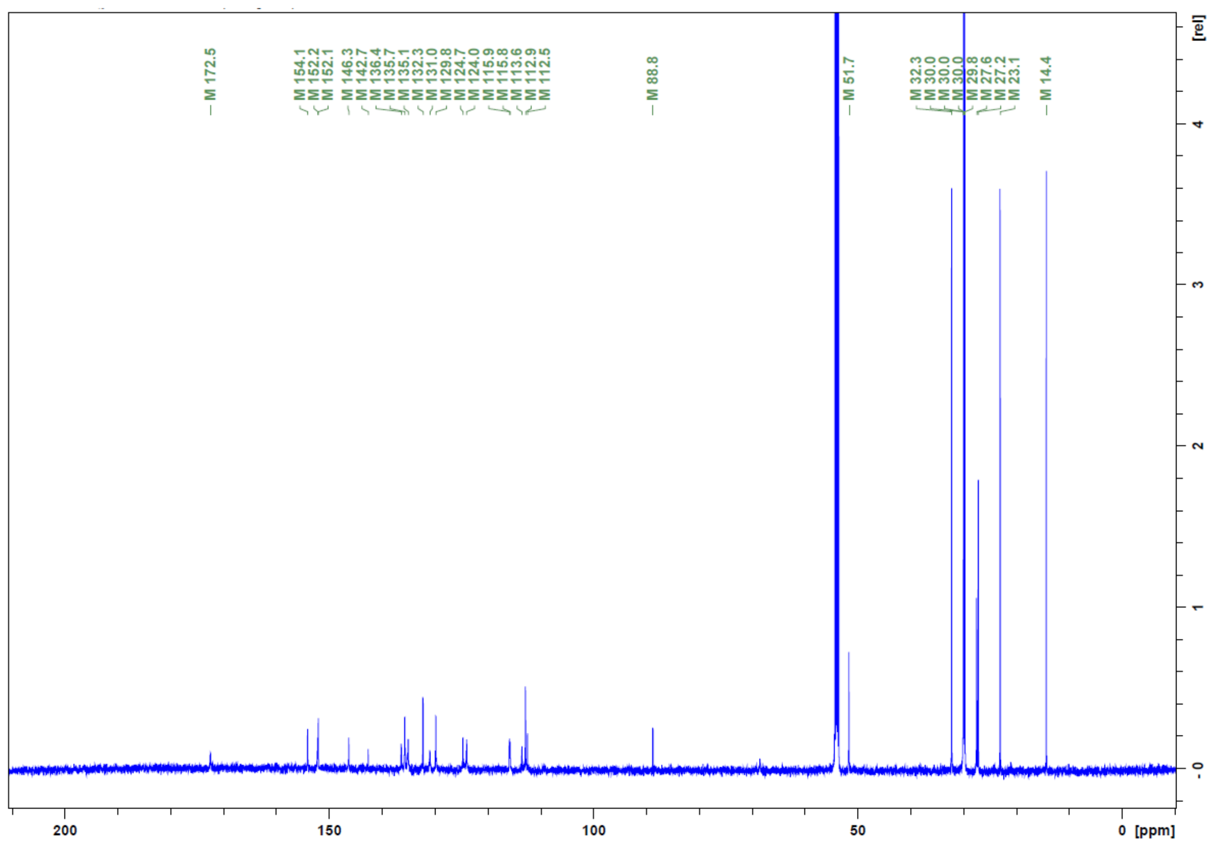

Figure S15. $^{13}C\{^{1}H\}$ NMR spectrum (151 MHz, CD_2Cl_2, 273K) of **TPE-TCNQ**.

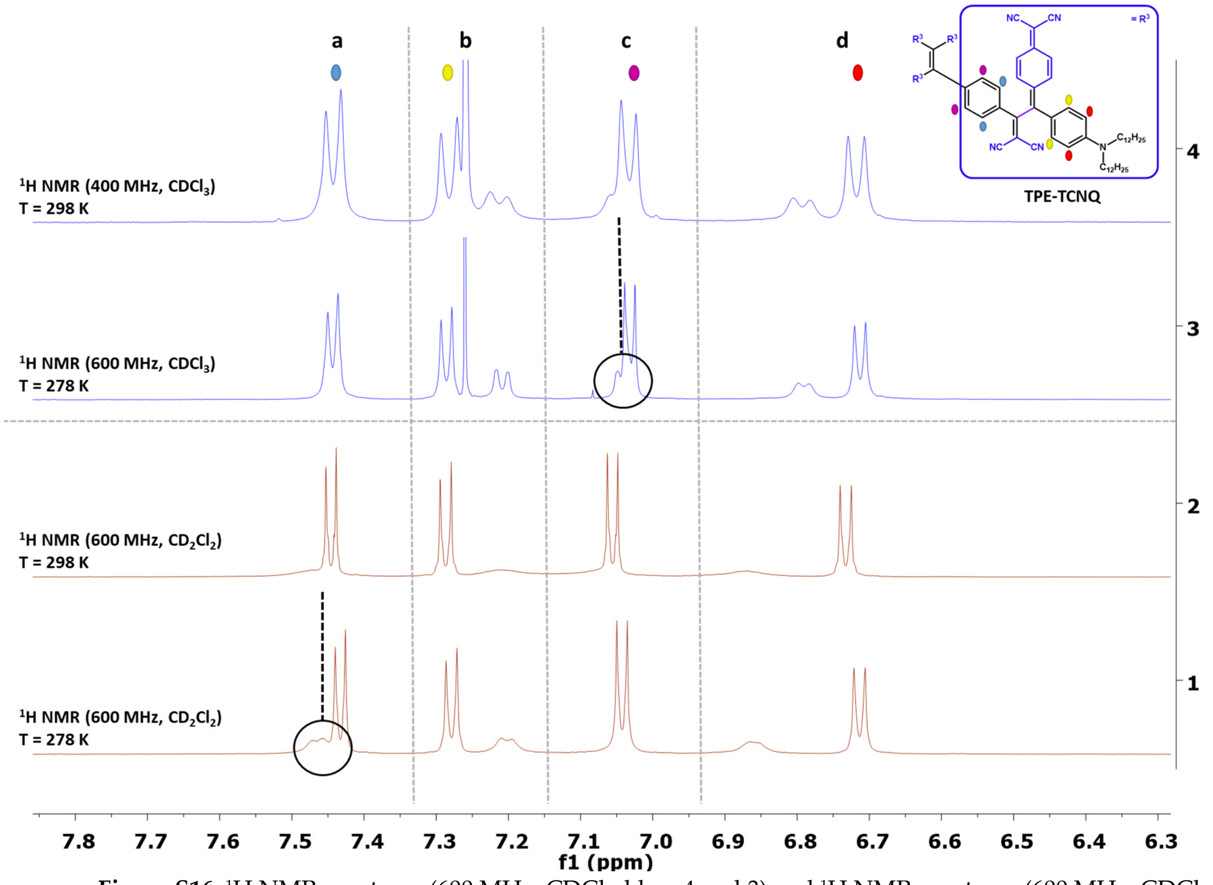

Figure S16. ^{1}H NMR spectrum (600 MHz, $CDCl_3$, blue, 4 and 3) and ^{1}H NMR spectrum (600 MHz, $CDCl_3$, red, 2 and 1) of **TPE-TCNQ** at 298 K and 278 K.

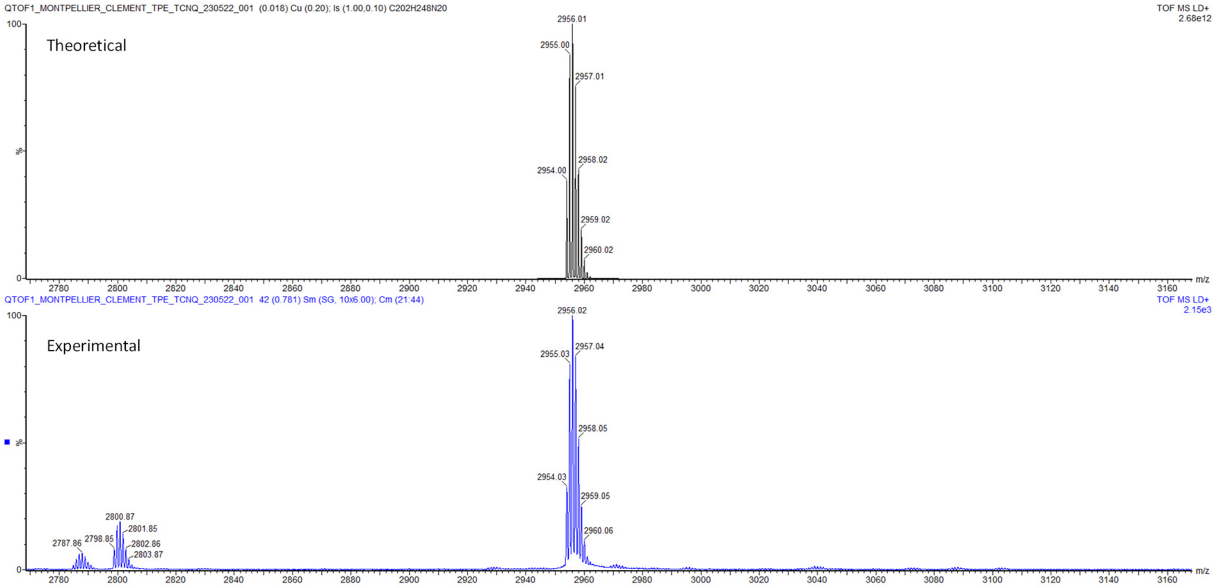

Elemental Composition Report

Single Mass Analysis
Tolerance = 25.0 PPM / DBE: min = -1.5, max = 200.0
Element prediction: Off
Number of isotope peaks used for i-FIT = 3

Monoisotopic Mass, Odd and Even Electron Ions
21 formula(e) evaluated with 2 results within limits (up to 50 closest results for each mass)
Elements Used:
C: 20-250 H: 0-250 N: 20-20

Minimum:				-1.5				
Maximum:			5.0	25.0	200.0			
Mass	Calc. Mass	mDa	PPM	DBE	i-FIT	Norm	Conf(%)	Formula
2953.9946	2954.0021	-7.5	-2.5	89.0	26.3	2.890	5.56	C202 H248 N20
	2953.9770	17.6	6.0	165.5	23.5	0.057	94.44	C213 H117 N20

Figure S17. High resolution MALDI-TOF mass spectrum of **TPE-TCNQ** (matrix: DCTB/NaI).

5. TPE-F4-TCNQ

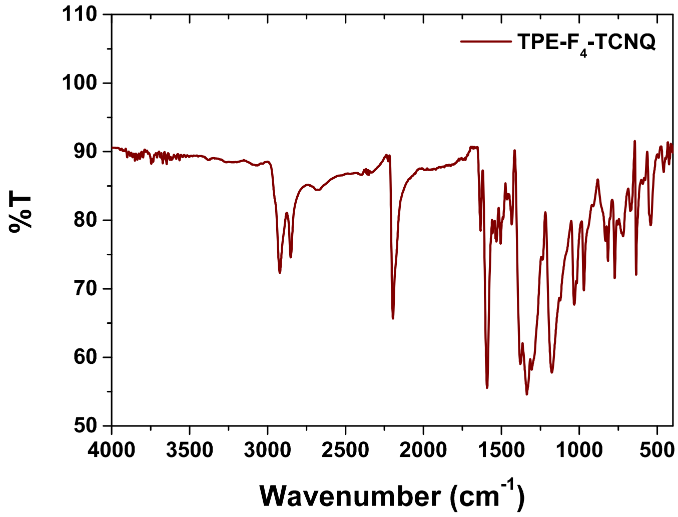

Figure S18. FTIR-ATR spectrum of **TPE-F4-TCNQ**.

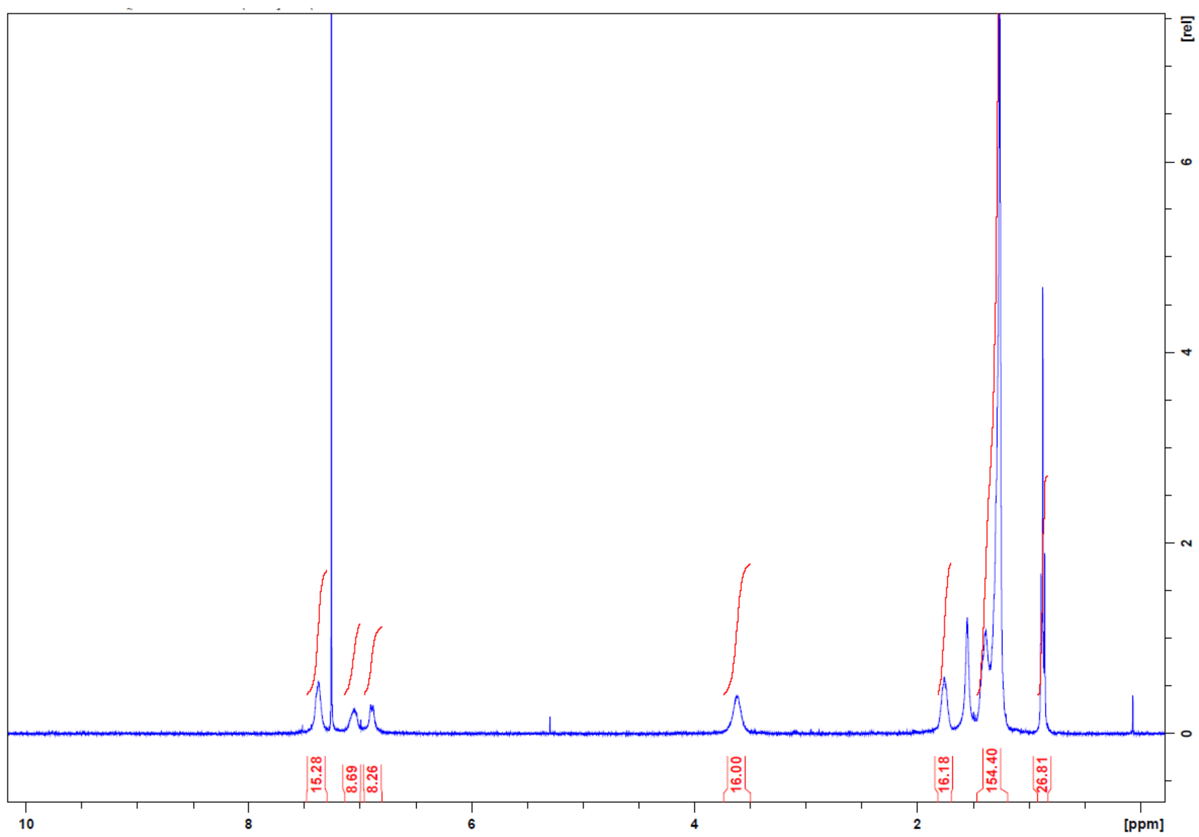

Figure S19. ^{1}H NMR (400 MHz, $CDCl_3$, 298K) of **TPE-F$_4$-TCNQ**.

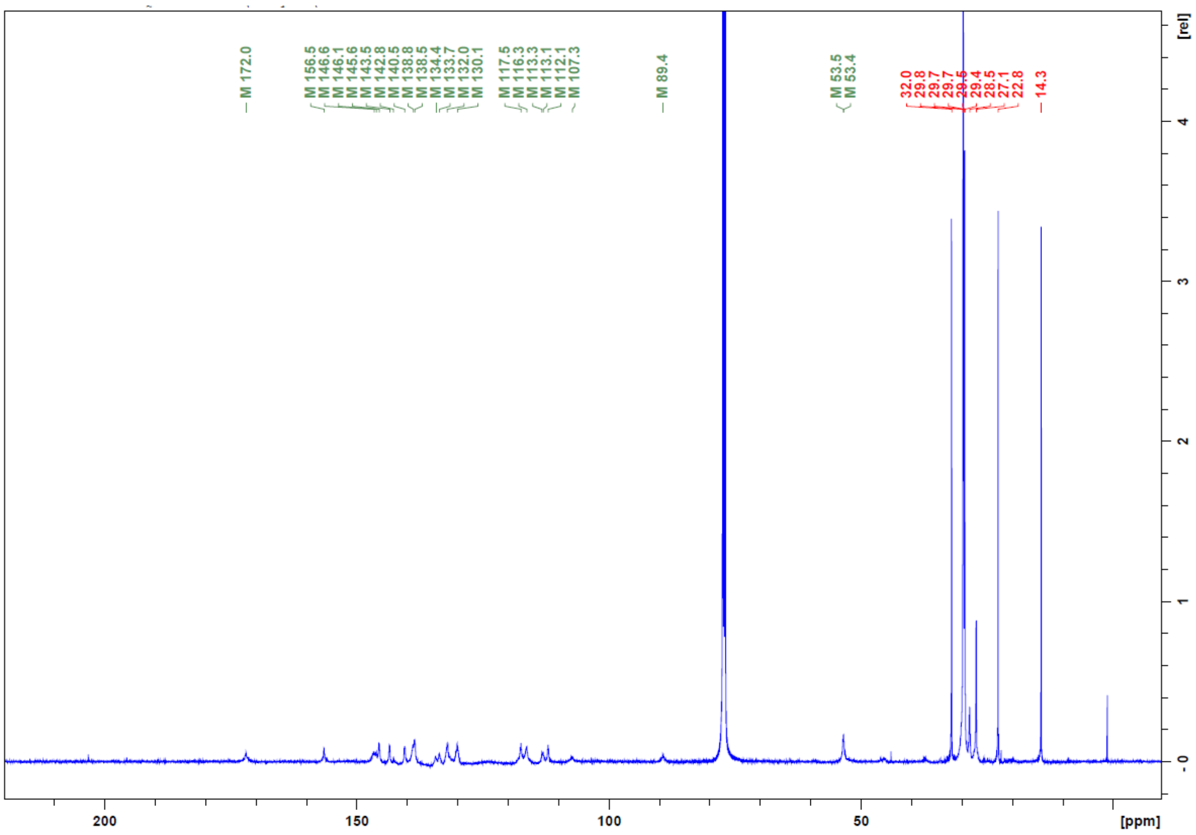

Figure S20. $^{13}C\{^1H\}$ NMR spectrum (126 MHz, $CDCl_3$, 298K) of **TPE-F$_4$-TCNQ**.

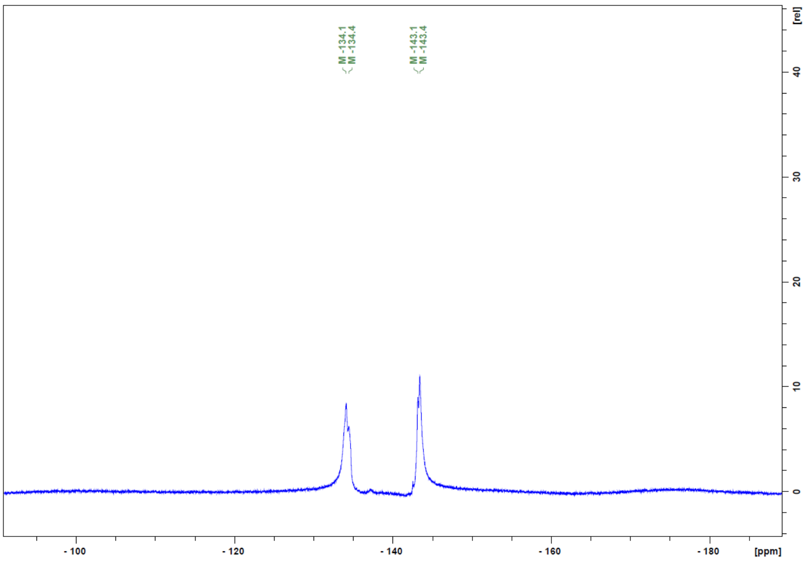

Figure S21. $^{19}F\{^{1}H\}$ NMR spectrum (376 MHz, $CDCl_3$, 298K) of **TPE- F_4-TCNQ**.

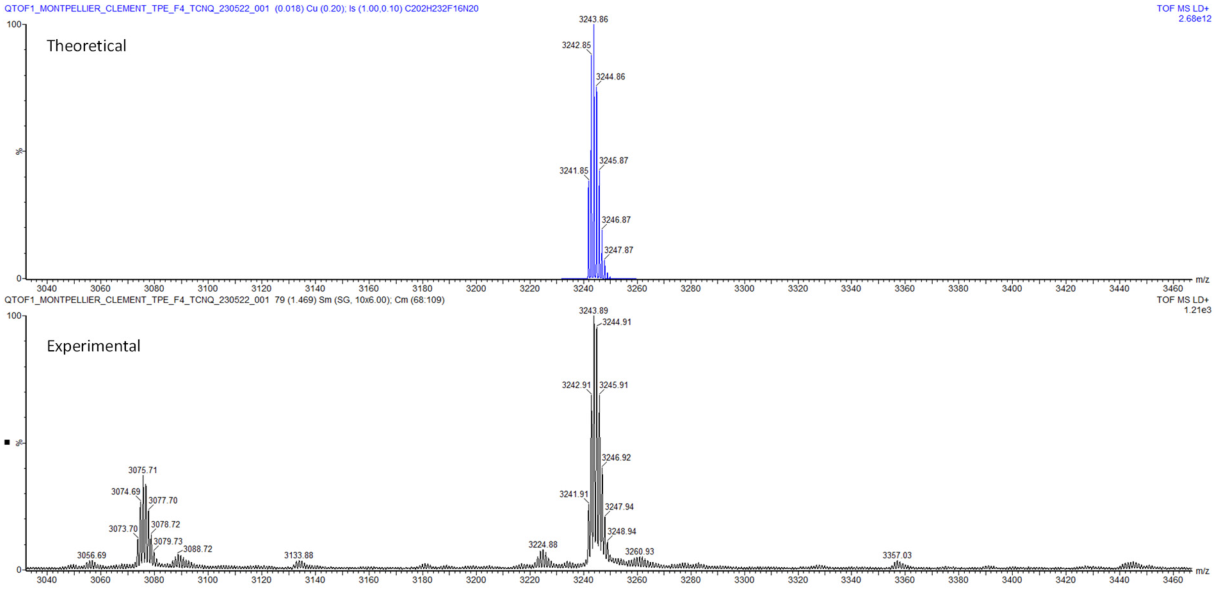

Elemental Composition Report

Single Mass Analysis
Tolerance = 25.0 PPM / DBE: min = -1.5, max = 200.0
Element prediction: Off
Number of isotope peaks used for i-FIT = 3

Monoisotopic Mass, Odd and Even Electron Ions
21 formula(e) evaluated with 3 results within limits (up to 50 closest results for each mass)
Elements Used:
C: 20-250 H: 0-250 N: 20-20 F: 16-16

Minimum:				-1.5				
Maximum:			5.0	25.0	200.0			
Mass	Calc. Mass	mDa	PPM	DBE	i-FIT	Norm	Conf(%)	Formula
3241.8586	3241.8513	7.3	2.3	89.0	21.5	1.084	33.83	C202 H232 N20 F16
	3241.8263	32.3	10.0	165.5	21.0	0.556	57.33	C213 H101 N20 F16
	3241.9202	-61.6	-19.0	158.5	22.9	2.426	8.84	C212 H113 N20 F16

Figure S22. High resolution MALDI-TOF mass spectrum of **TPE-F_4-TCNQ** (matrix: DCTB/NaI).

II. Absorption and fluorescence spectroscopies

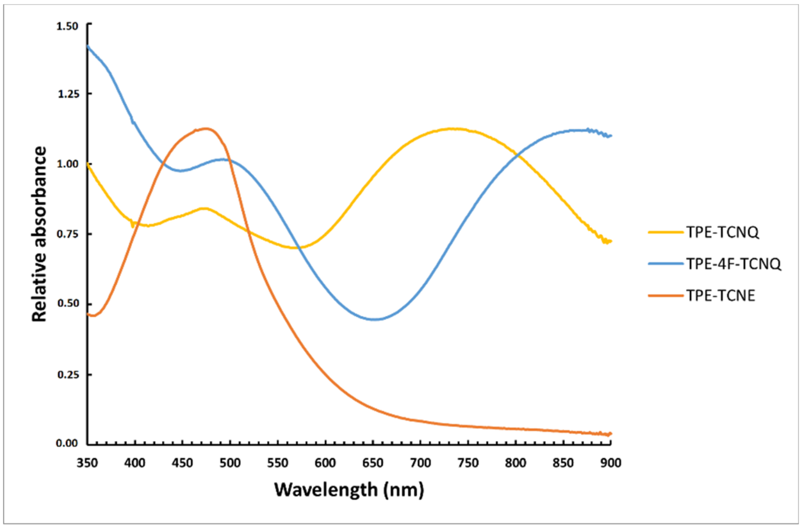

Figure S23. Absorption spectra of TPE adducts as thin films on quartz substrate.

III. Study of AIE behavior

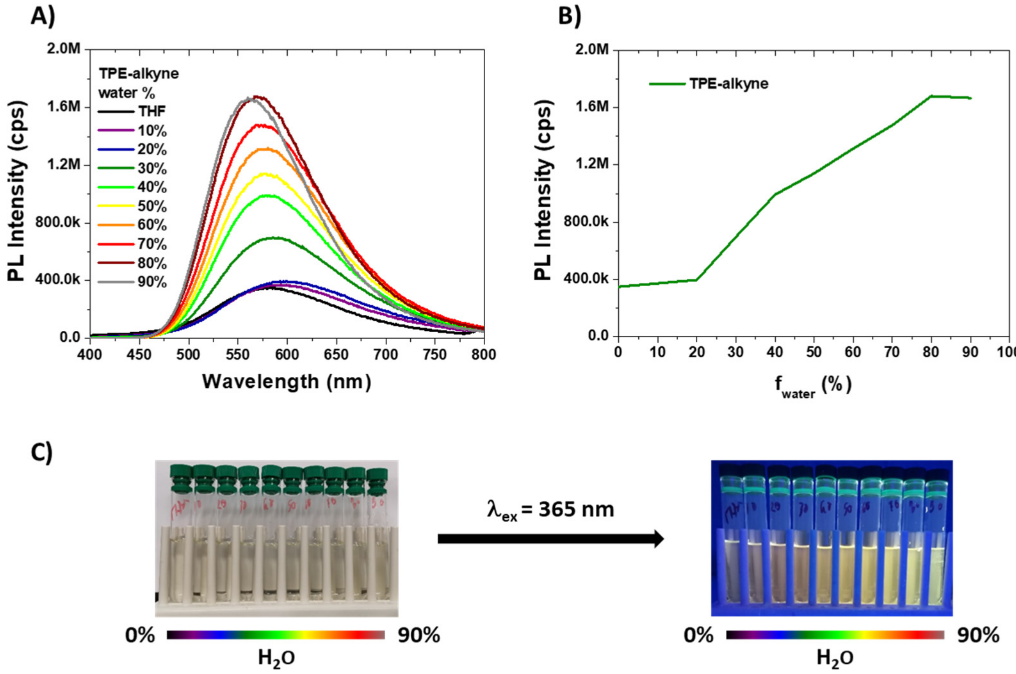

Figure S24. A) emission spectra ($\lambda_{exc.}$ = 360 nm) of **TPE-Alkyne** in various THF / water mixtures (C = 6.10^{-6} mol.L^{-1}). ; B) variation of the relative emission intensity vs. water fraction (I_{THF}: Intensity of the fluorescence of **TPE-alkyne** at the maximum emission in THF and I: Intensity of the fluorescence of TPE-alkyne at the maximum emission in various THF /water mixtures) and C) photographs of the corresponding mixtures under natural (left) and UV (left, 365 nm) light illumination.

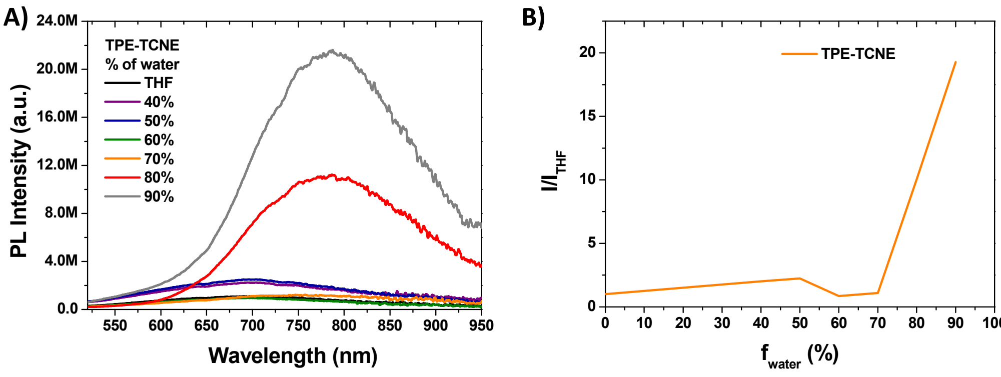

Figure S25. A) emission spectra ($\lambda_{exc.}$ = 480 nm) of **TPE-TCNE** in various THF / water mixtures (C = 5.3.10^{-5} mol.L^{-1}) ; B) variation of the relative emission intensity vs. water fraction.

IV. pH-dependent emission studies

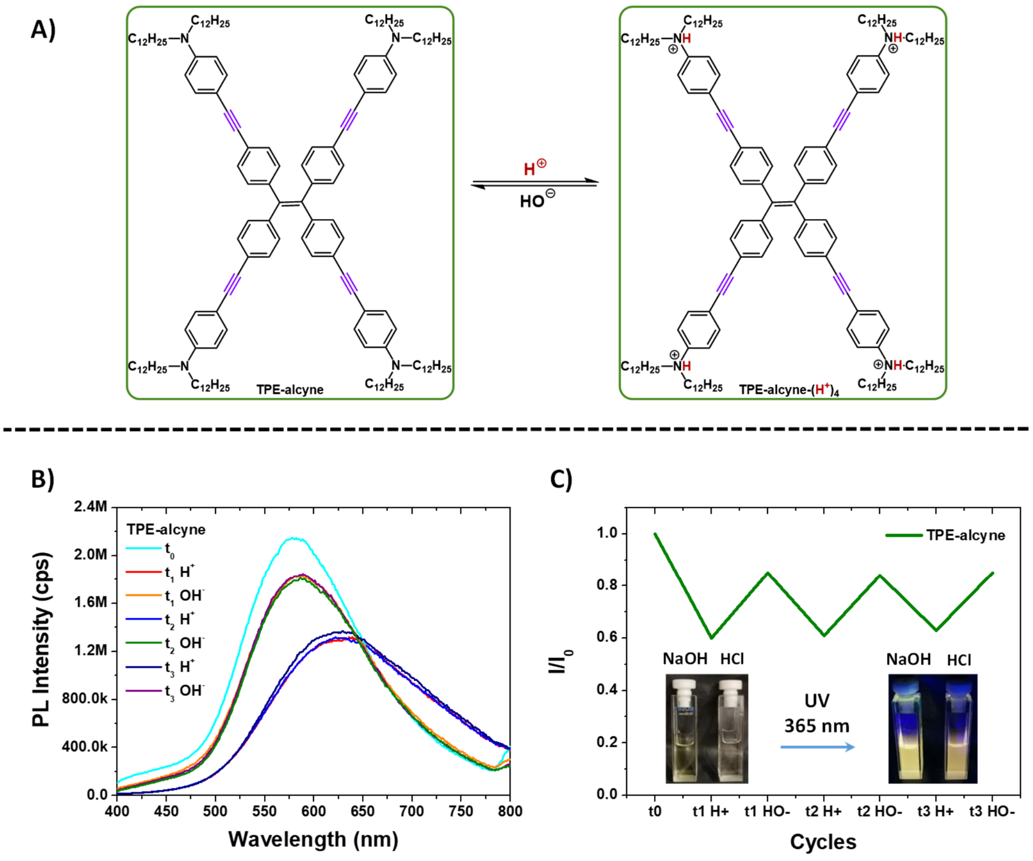

Figure S26. A) Reversible proposed acidification scheme for **TPE-alkyne** in THF/water solution ; B) variation of the emission spectra as a function of the pH (H^+ : addition of an aqueous HCl solution up to pH = 1 +/- 0.1 and OH^- : addition of an aqueous NaOH solution up to pH = 13 +/- 0.1) (C = 1.10^{-5} mol.L^{-1}, $\lambda_{exc.}$ = 360 nm) and C) drawing of the relative emission intensity variation upon pH cycling and corresponding images under natural and UV (365 nm) light illumination.

V. Electrochemistry

1. TPE-TCNE

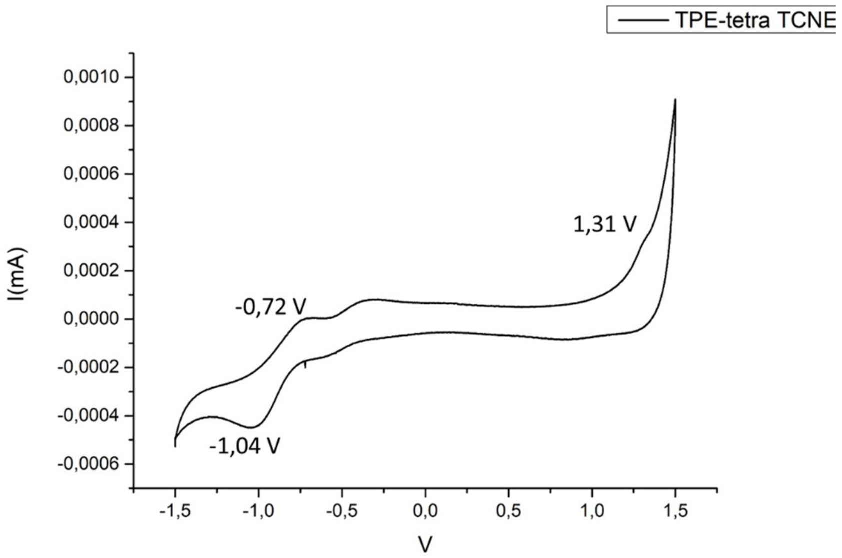

Figure S27. Cyclic voltammogram of **TPE-TCNE** in 0.2 M solution of Bu_4NPF_6 in CH_2Cl_2 (scan rate: 100 mV.s^{-1}; . electrode potential vs. SCE (saturated calomel electrode)).

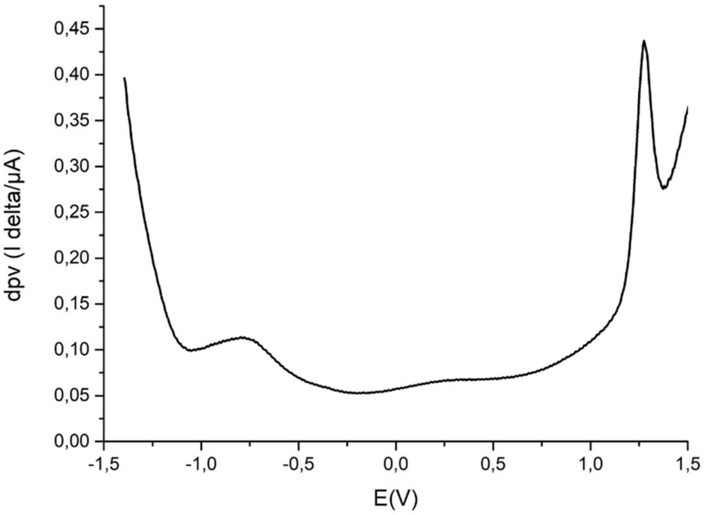

Figure S28. Differential pulse voltammetry (DPV) of **TPE-TCNE** in 0.2 M solution of Bu_4NPF_6 in CH_2Cl_2 (scan rate: 100 mV.s^{-1}; electrode potential vs. SCE).

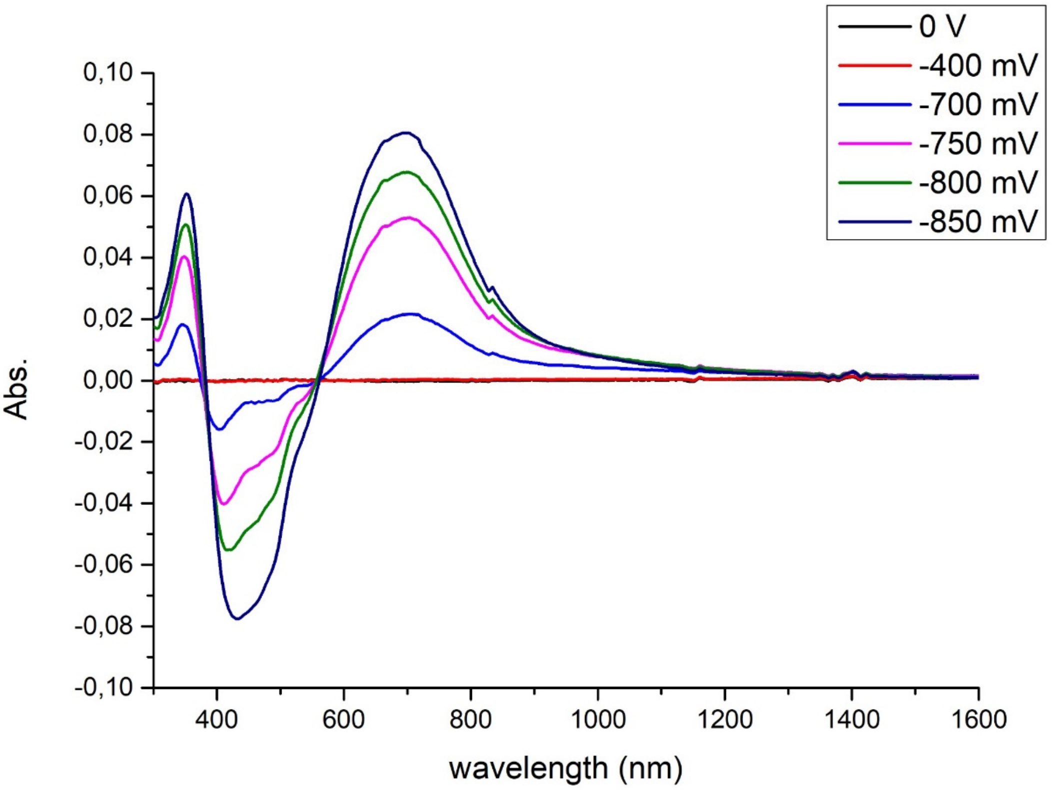

Figure S29. Differential absorption spectra obtained during the reduction of **TPE-TCNE** in 0.2 M solution of Bu_4NPF_6 in CH_2Cl_2 (scan rate: 100 mV.s^{-1}; electrode potential vs. SCE).

2. TPE-TCNQ

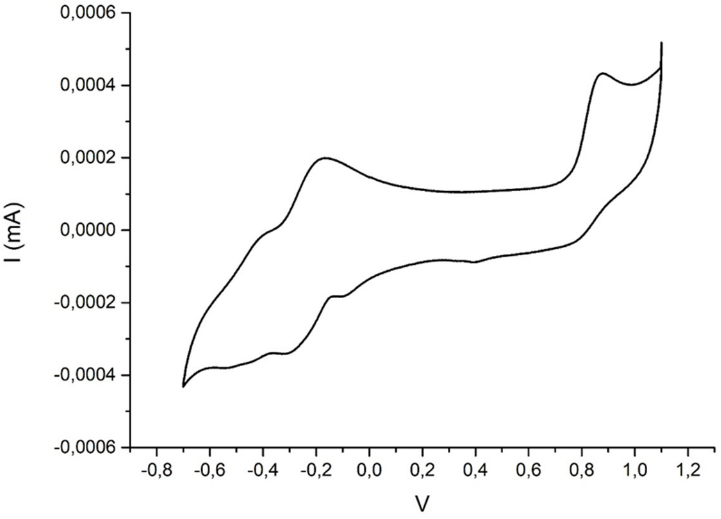

Figure S30. Cyclic voltammogram of **TPE-TCNQ** in 0.2 M solution of Bu_4NPF_6 in CH_2Cl_2 (scan rate: 100 mV.s^{-1}; electrode potential vs. SCE).

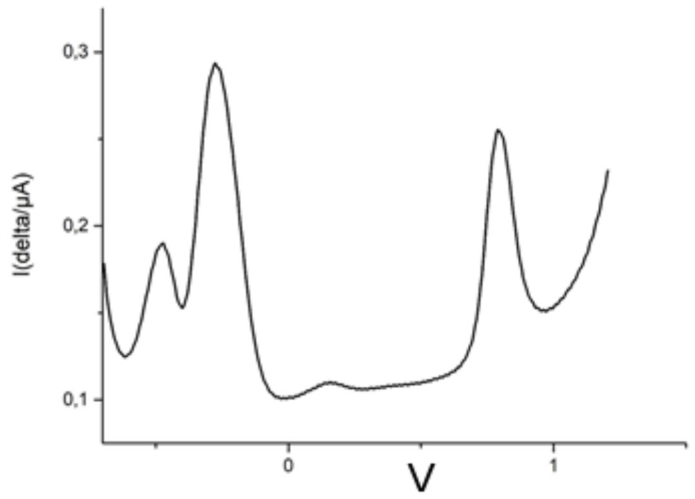

Figure S31. Differential pulse voltammetry (DPV) of **TPE-TCNQ** in 0.2 M solution of Bu_4NPF_6 in CH_2Cl_2 (scan rate: 100 $mV.s^{-1}$; electrode potential vs. SCE).

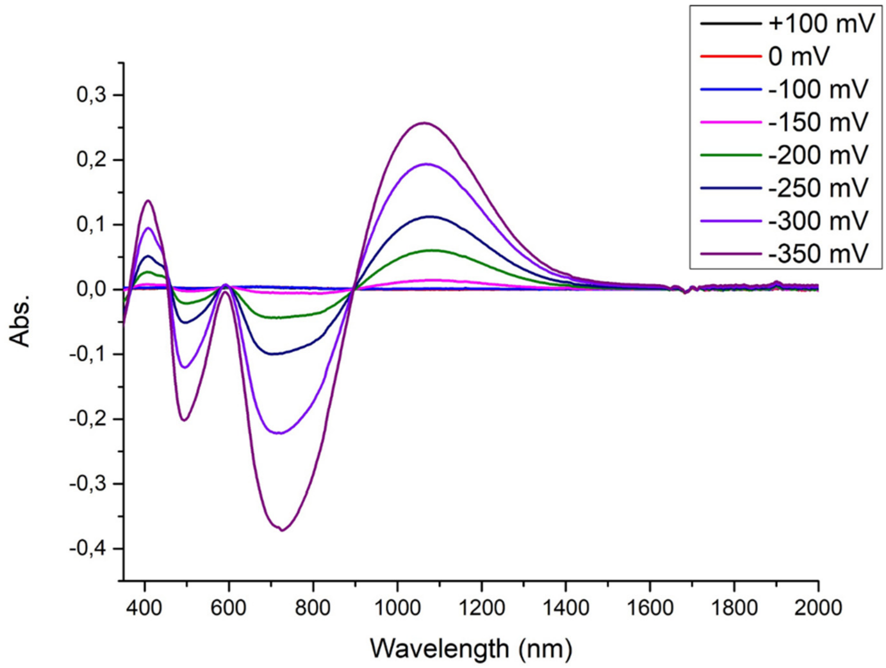

Figure S32. Differential absorption spectra obtained during the reduction of **TPE-TCNQ** in 0.2 M solution of Bu_4NPF_6 in CH_2Cl_2.

3. TPE-F_4-TCNQ

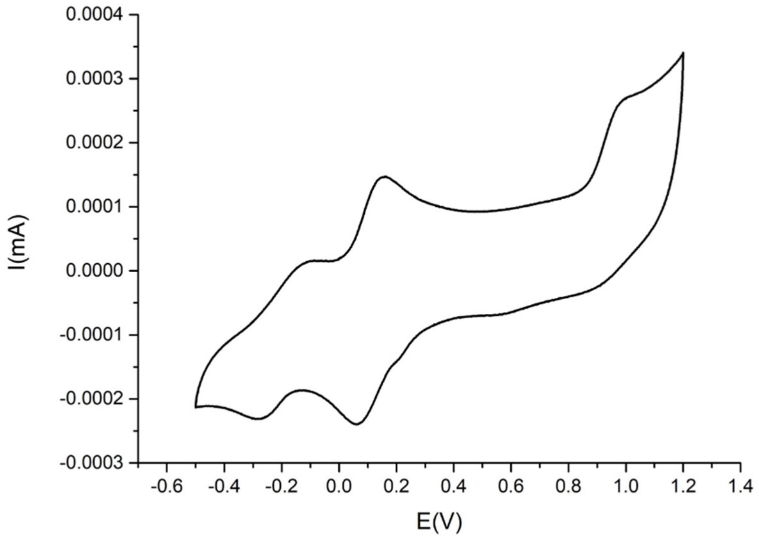

Figure S33. Cyclic voltammogram of **TPE-F_4-TCNE** in 0.2 M solution of Bu_4NPF_6 in CH_2Cl_2 (scan rate: 100 mV.s^{-1}; electrode potential vs. SCE).

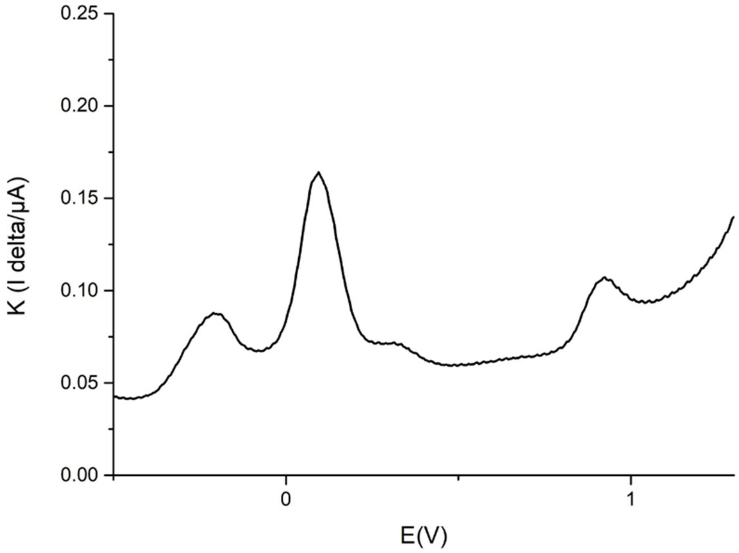

Figure S34. Differential pulse voltammetry (DPV) of **TPE-F_4-TCNQ** in 0.2 M solution of Bu_4NPF_6 in CH_2Cl_2 (scan rate: 100 mV.s^{-1}; electrode potential vs. SCE).

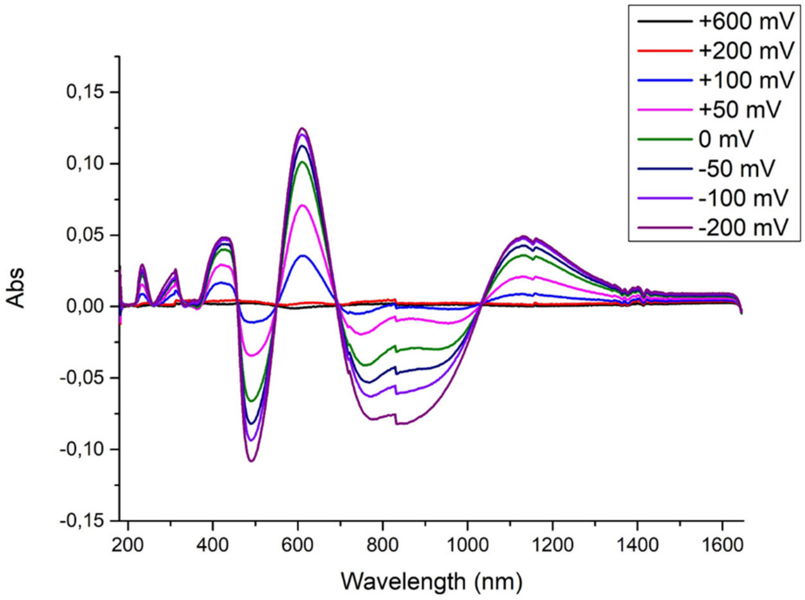

Figure S35. Differential absorption spectra obtained during the reduction of **TPE-F$_4$-TCNE** in 0.2 M solution of Bu_4NPF_6 in CH_2Cl_2.

VI. Thermal and photothermal studies

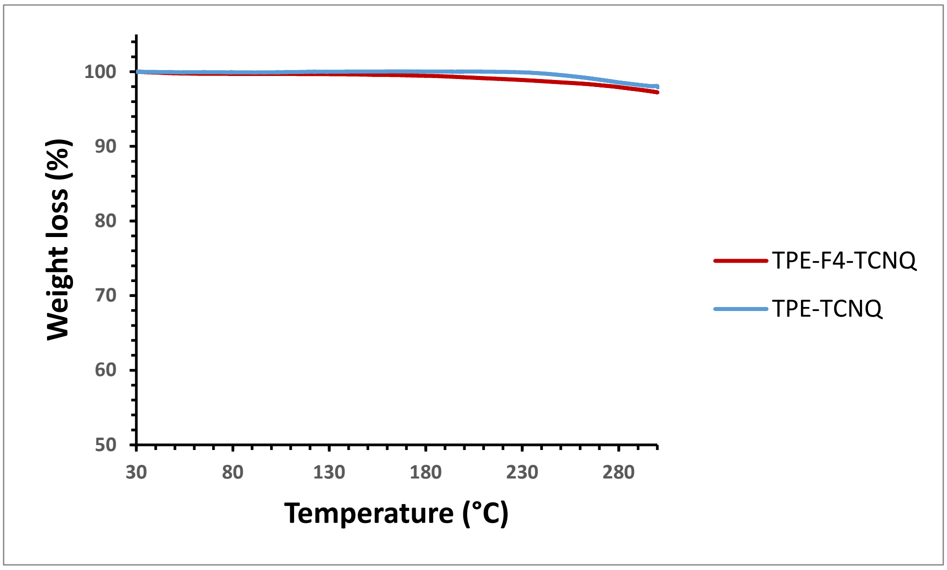

Figure S36. Thermogravimetric analysis (TGA) of **TPE-TCNQ** (blue) and **TPE-F$_4$-TCNQ** (red) under N_2 flow.

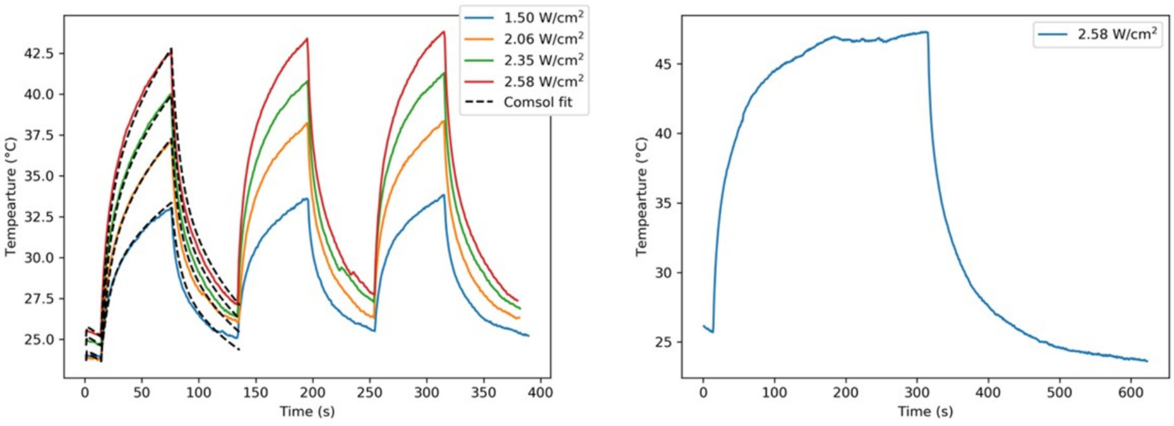

Figure S37. Photothermal conversion behavior of **TPE-TCNQ** as a thin film under 808 nm laser irradiation at different laser powers (1.50, 2.06, 2.35, and 2.58 cm^{-2}) vs. Comsol fits.

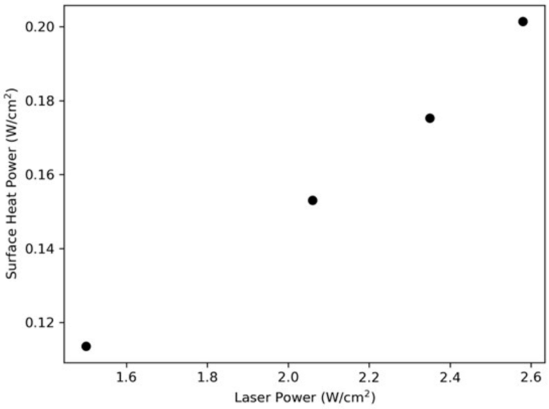

Figure S38. Surface heat power vs. laser power at 808 nm for **TPE-TCNQ** as a thin film.

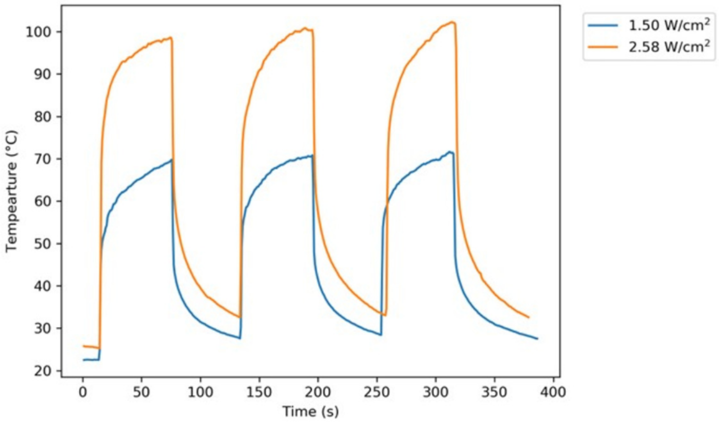

Figure S39. Photothermal conversion behavior of **TPE-TCNQ** as a powder under 808 nm laser irradiation at laser powers of 1.50 and 2.58 cm^{-2}.

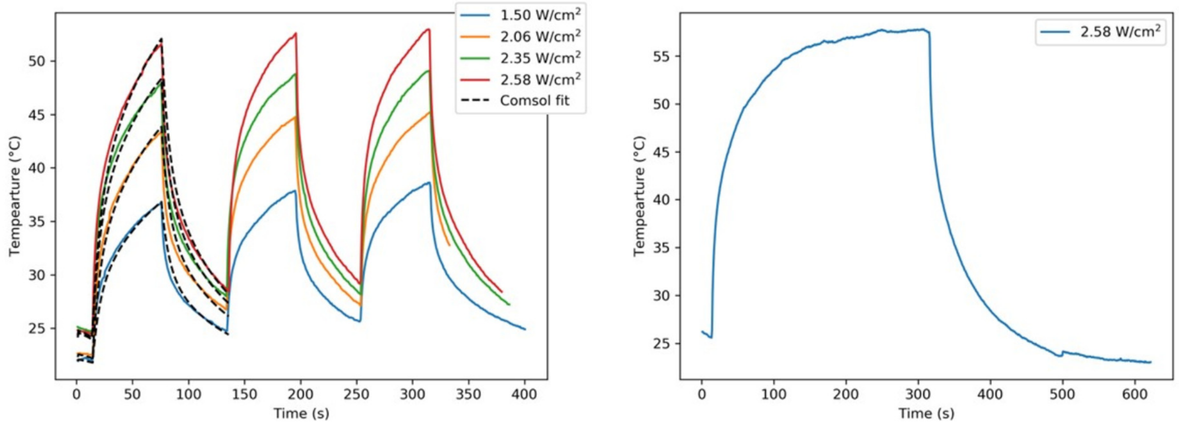

Figure S40. Photothermal conversion behavior of **TPE-F$_4$-TCNQ** as a thin film under 808 nm laser irradiation at different laser powers (1.50, 2.06, 2.35, and 2.58 cm^{-2}) vs. Comsol fits.

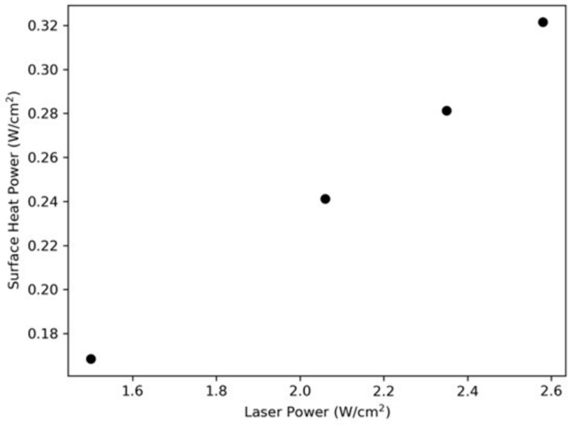

Figure S41. Surface heat power vs. laser power at 808 nm for **TPE-TCNQ** as a thin film.

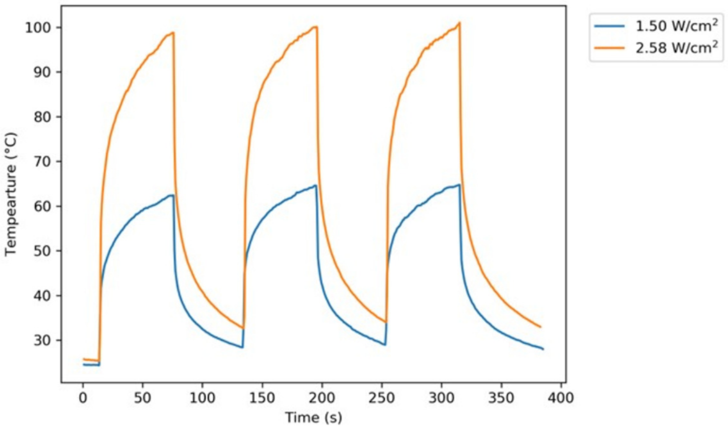

Figure S42. Photothermal conversion behavior of **TPE-F$_4$-TCNQ** as a powder under 808 nm laser irradiation at laser powers of 1.50 and 2.58 cm^{-2}.

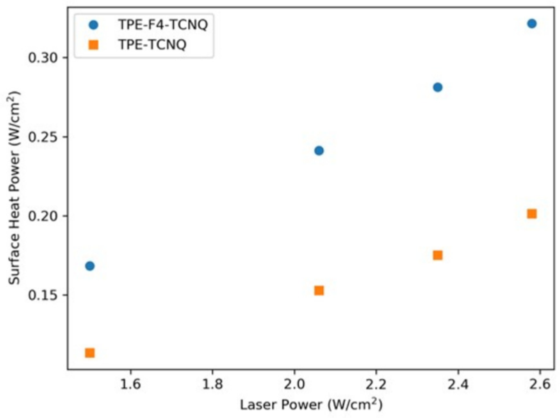

Figure S43. Comparison of the surface heat power vs. laser power at 808 nm for **TPE-TCNQ** (orange) and **TPE-F_4-TCNQ** as thin films.

VII. Calculations

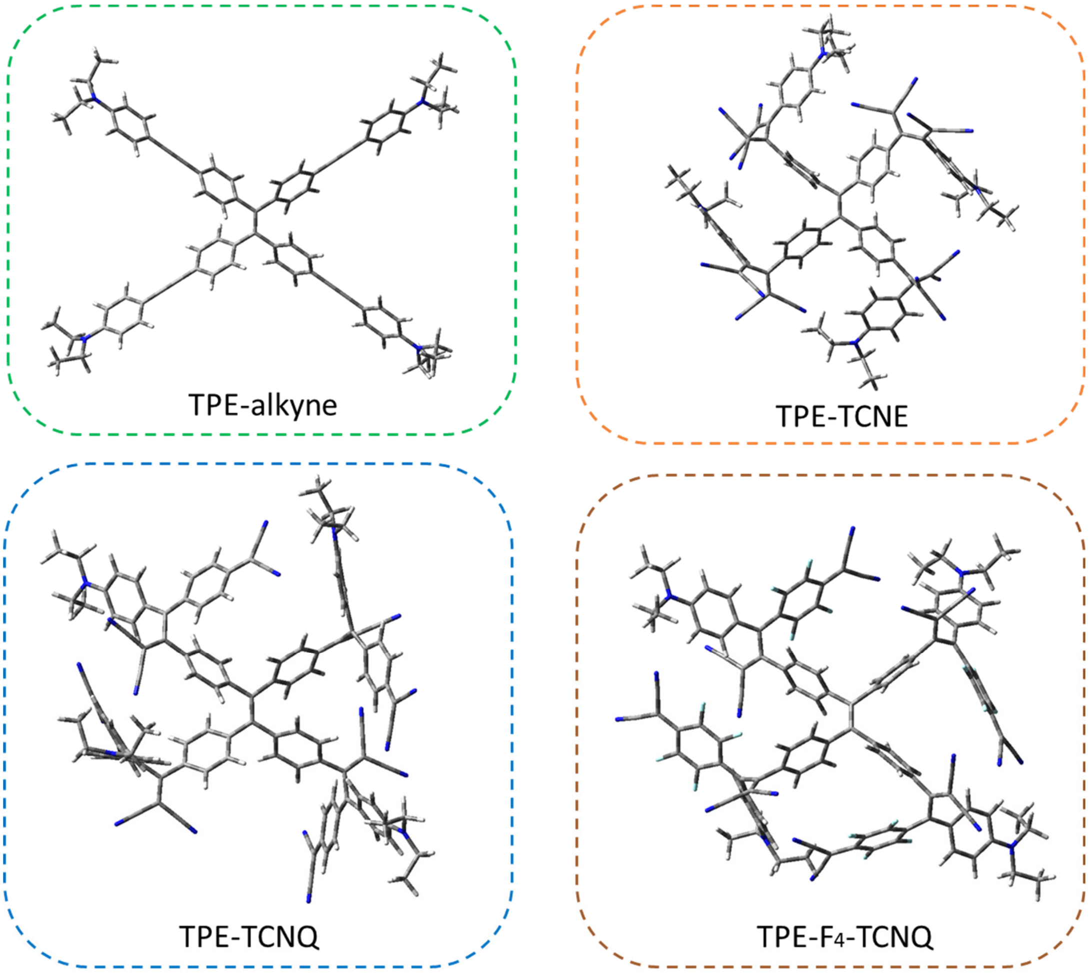

Figure S44. Optimized geometry of the investigated compounds computed at DFT-Cam-B3LYP/6-311G(d,p) theory level.

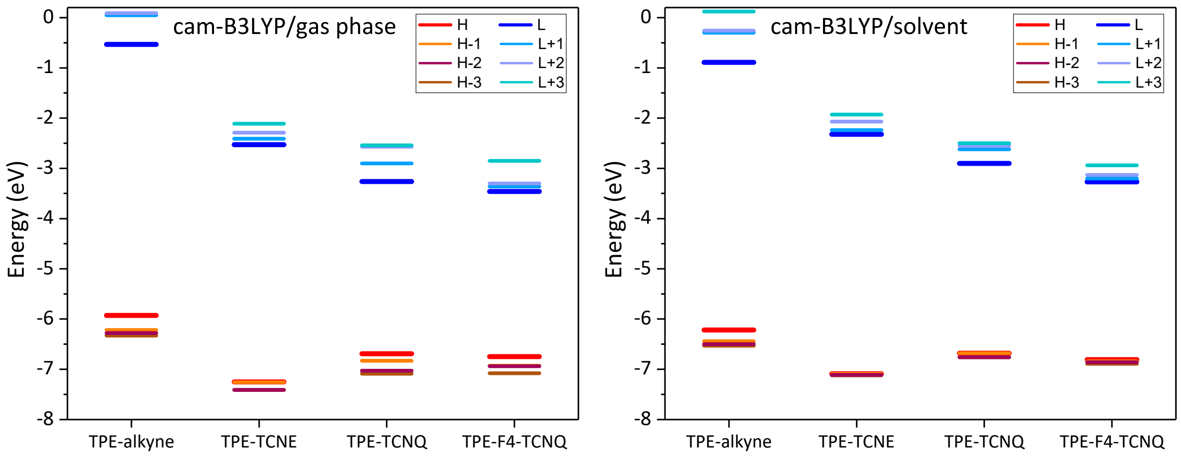

Figure S45. Energy alignment of the four frontier occupied and virtual molecular orbitals for the investigated systems in both gas phase and in THF/DCM solution, as calculated at CAM-B3LYP/6- 311G (d,p) level.

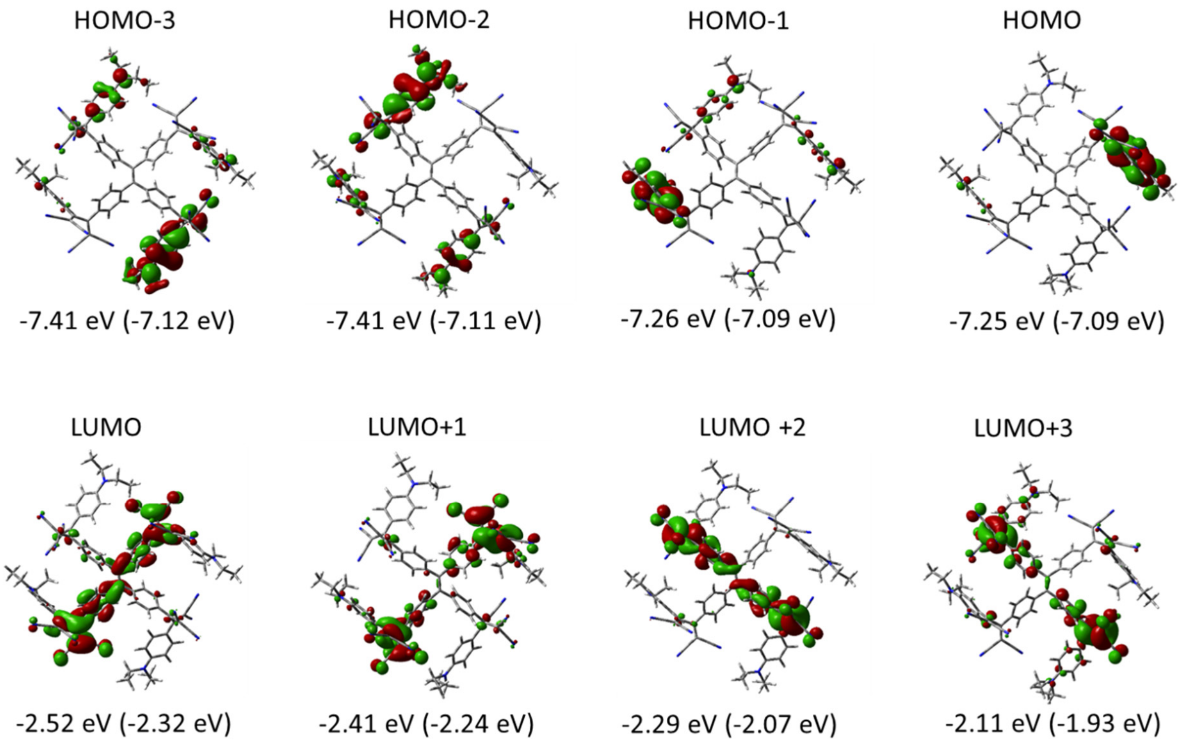

Figure S46. Shape of the four occupied and virtual molecular orbitals for **TPE-TCNE** in the gas phase. Energies are computed at the CAM-B3LYP/6-311G(d,p) level of theory in gas phase and in THF solution. Isovalues are set to 0.02 e/au^3.

Table S1: Assignment of electronic excitations for the investigated compounds, as obtained at the TD-DFT/Cam-B3LYP level [a].

Transition number	Computed transition energy λ, nm (eV)	Oscillator strength	Main contributions
		**TPE-alkyne**	
1	367 (3.38)	2.02	H->L (67%), H-4->L (12%)
3	330 (3.76)	4.82	H->L+1 (33%), H-2->L (30%), H-3->L+2
5	287 (4.32)	0.54	H-4->L (22%), H-2->L+1 (22%), H-1->L+2
		**TPE-TCNE**	
1	443 (2.80)	0.20	H-1->L+1 (35%), H-1->L (33%), H->L (15%)
2	443 (2.80)	0.28	H->L+1 (46%), H->L (25%), H-1->L (15%)
3	401 (3.09)	0.24	H-2->L+5 (26%), H-2->L+3 (25%), H-3->L+4
4	399 (3.11)	0.95	H-3->L+4 (28%), H-3->L+3 (24%), H-2->L+4
		**TPE-TCNQ**	
1	625 (1.98)	0.87	H→L (87%)
2	549 (2.26)	0.39	H-2->L+1 (31%), H-1->L+2 (31%), H-3->L+1
3	545 (2.27)	1.75	H-1->L+2 (50%), H-2->L+1 (18%), H-3-
4	518 (2.39)	1.29	H-3->L+3 (38%), H-2->L+3 (37%), H-1->L+2
		**TPE-F_4-TCNQ**	
1	675 (1.84)	0.10	H->L (46%), H-1->L (20%), H-2->L+1 (12%)
2	642 (1.93)	2.16	H-2->L+1 (62%), H->L (16%)
3	606 (2.04)	1.81	H-3->L+2 (73%), H-2->L+1 (12%)
4	585 (2.12)	0.69	H-1->L+3 (50%), HOMO->L+3 (30%)

[a] Only excitations with a significant oscillator strength around the main peak of the lowest absorption band are included. H stands for HOMO and L for LUMO

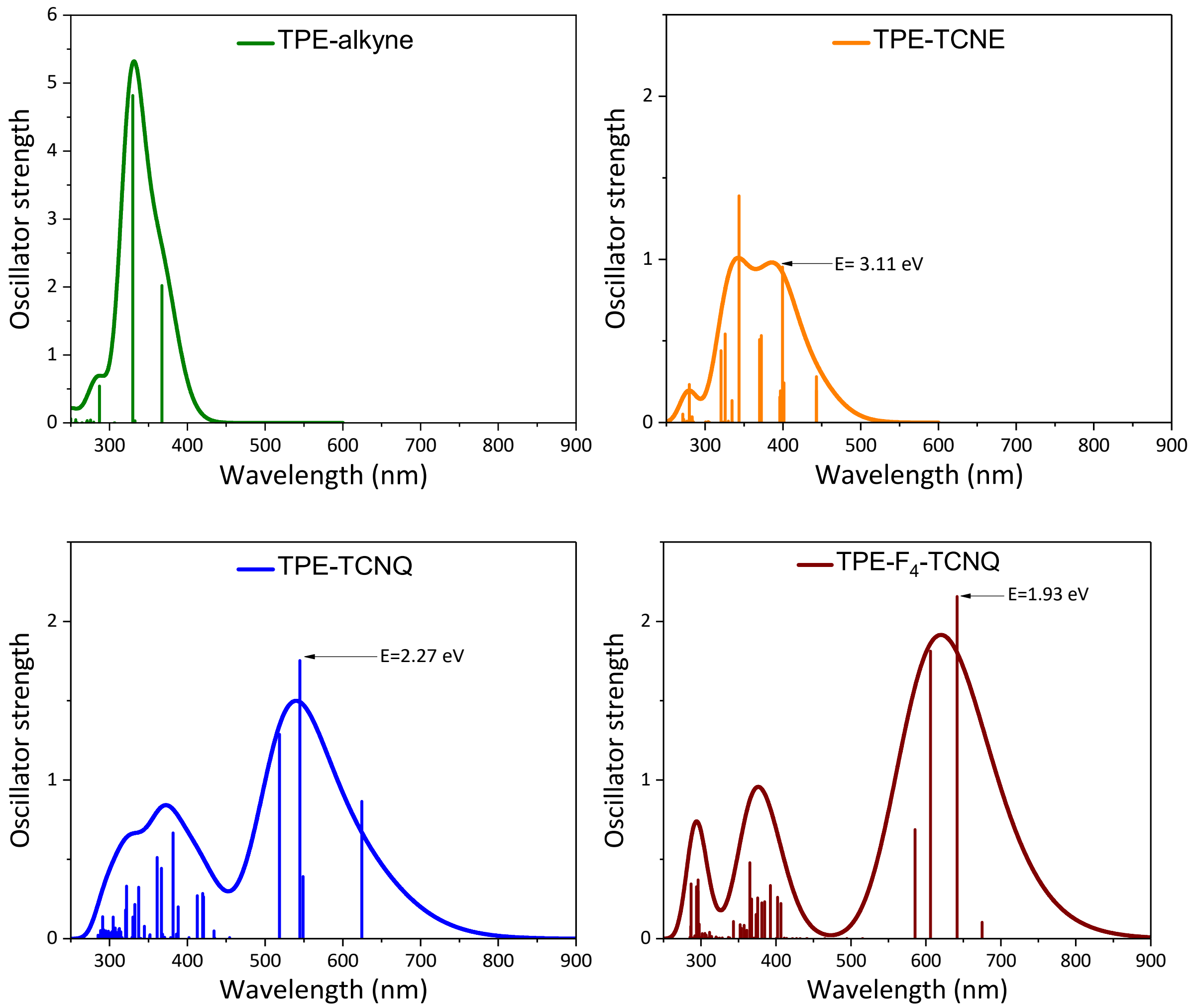

Figure S47. Simulated UV-Vis absorption spectra of the investigated compounds in THF solution at the TDDFT/Cam-B3LYP level with all transition peaks. The main transition energy of the CT absorption band for TPE adducts is also pointed with an arrow.
